# Functional neuroimaging of visual creativity: a systematic review and meta‐analysis

**DOI:** 10.1002/brb3.540

**Published:** 2016-08-11

**Authors:** Laura M. Pidgeon, Madeleine Grealy, Alex H. B. Duffy, Laura Hay, Chris McTeague, Tijana Vuletic, Damien Coyle, Sam J. Gilbert

**Affiliations:** ^1^ School of Psychological Sciences and HealthUniversity of StrathclydeGlasgowUK; ^2^ Department of Design, Manufacture and Engineering ManagementUniversity of StrathclydeGlasgowUK; ^3^ Intelligent Systems Research CentreUniversity of UlsterDerryNorthern Ireland; ^4^ Institute of Cognitive NeuroscienceUniversity College LondonLondonUK

**Keywords:** creative cognition, creative ideation, electroencephalography, functional magnetic resonance imaging, idea generation, ideation, visual creativity, visual design, visual imagery

## Abstract

**Introduction:**

The generation of creative visual imagery contributes to technological and scientific innovation and production of visual art. The underlying cognitive and neural processes are, however, poorly understood.

**Methods:**

This review synthesizes functional neuroimaging studies of visual creativity. Seven functional magnetic resonance imaging (fMRI) and 19 electroencephalography (EEG) studies were included, comprising 27 experiments and around 800 participants.

**Results:**

Activation likelihood estimation meta‐analysis of the fMRI studies comparing visual creativity to non‐rest control tasks yielded significant clusters in thalamus, left fusiform gyrus, and right middle and inferior frontal gyri. The EEG studies revealed a tendency for decreased alpha power during visual creativity compared to baseline, but comparisons of visual creativity to non‐rest control tasks revealed inconsistent findings.

**Conclusions:**

The findings are consistent with suggested contributions to visual creativity of prefrontally mediated inhibition, evaluation, and working memory, as well as visual imagery processes. Findings are discussed in relation to prominent theories of the neural basis of creativity.

## 1 Introduction

Creative ideation, the generation of novel and useful ideas (Runco & Jaeger, 2012; Stein, 1953), is critical to the advancement of scientific and technological innovation, and to artistic, musical, and literary endeavors (e.g., Dietrich & Kanso, 2010; Fink et al., 2009). Its adaptive value in enabling responses to novel, infrequent events has also been noted (Jung, 2014). Visual creativity refers to the generation of novel and useful mental visual imagery, which may lead to the production of novel and useful visual forms (e.g., sketches, paintings) (Aziz‐Zadeh, Liew & Dandekar, 2013; Dake, 1991; Runco and Jaeger, 2012). According to Runco and Jaeger's (2012) standard definition of creativity, “useful” refers to outputs that are effective or valuable in accordance with the task demands—as such, this definition can encompass tasks emphasizing the functionality, esthetics, or originality of solutions (e.g., Ellamil, Dobson, Beeman, & Christoff, 2012; Petsche, 1996). In design, visual creativity is a key component in the generation of mental images and sketches of novel and functional products (Fish & Scrivener, 1990), while in an artistic context, the esthetics of visual creative solutions are highlighted, visual creativity has significant cultural importance (Damasio, 2001).Visual creativity contrasts with nonvisual creativity, where novel and useful outputs in verbal, literary, or musical domains are produced (e.g., Boccia, Piccardi, Palermo, Nori, & Palmiero, 2015). Despite this distinction, creative visual imagery is thought to be a component process of creative ideation generally, including nonvisual creativity (e.g., Abraham, 2013; Abraham & Windmann, 2007; Finke, 1996, 2014).

Despite the contribution of visual creativity to innovation in many domains, the underlying cognitive and neural processes remain poorly specified. An understanding of these processes may inform future studies evaluating training of the appropriate cognitive skills, or neurofeedback interventions encouraging processes associated with successful visual creativity (e.g., Gruzelier, 2014). This is particularly important in populations in which visual creativity is of professional, social, or recreational value.

Cognitive models of creativity propose that creative ideation involves retrieval of semantic associations and their conceptual combination (Abraham, 2014; Benedek & Neubauer, 2013; Boden, 2004; Mednick, 1962; Mumford, Medeiros, & Partlow, 2012), as well as executive functions including inhibition of irrelevant responses (Benedek et al., 2014; Oberauer, Süβ, Wilhelm, & Wittmann, 2008), and response evaluation (Mumford et al., 2012; Sowden, Pringle, & Gabora, 2015). Independent contributions of associative and executive abilities have been supported in behavioral investigations of divergent thinking (Beaty, Silvia, Nusbaum, Jauk, & Benedek, 2014; Benedek & Neubauer, 2013). Proposed contributions of visual imagery to creativity have been supported by findings of positive associations between visual imagery ability and visual and verbal creative ability (Finke, 1996; González, Campos, & Pérez, 1997; Kozhevnikov, Kozhevnikov, Yu, & Blazhenkova, 2013; see LeBoutillier & Marks, 2003, for meta‐analysis; Palmiero, Cardi, & Belardinelli, 2011; cf. Antonietti, Bologna, & Lupi, 1997).

Semantic memory retrieval, visual imagery, inhibition, and evaluation are involved in many distinct tasks, not just in visual creativity. This highlights the need for careful selection of well‐matched control tasks in neuroimaging investigations of this ability. Control tasks involving similar or overlapping processes to visual creative tasks facilitate examination of the brain regions and cognitive processes that may be engaged to a relatively greater degree in tasks drawing on visual creativity (Abraham, 2014). Visual creativity is thought to differ from nonvisual creativity (e.g., generation of verbal or musical creative outputs), and visual noncreative tasks (e.g., generation of mental imagery from memory) in which visual image generation, manipulation, and evaluation are engaged to a greater extent (Finke, 1996; Gansler et al., 2011; Kozhevnikov et al., 2013; Palmiero, Nori, Aloisi, Ferrara, & Piccardi, 2015). Based on previous neuroimaging studies of visual imagery, visual creativity may engage regions linked to these functions, including early visual cortex, fusiform, V5/MT, posterior parietal cortex, and bilateral inferior frontal cortex (Kosslyn & Thompson, 2003; Mazard, Tzourio‐Mazoyer, Crivello, Mazoyer, & Mellet, 2004; see Tomasino & Gremese, 2015, for meta‐analysis). As imagery is proposed to contribute to visual creativity in combination with semantic associative and executive processes, visual creativity may be expected to engage regions associated with visual representation of semantic concepts (e.g., left fusiform; Kan, Barsalou, Olseth Solomon, Minor, & Thompson‐Schill, 2003), and top‐down modulation of visual regions involved in imagery (e.g., frontal operculum; Stokes, Thompson, Cusack, & Duncan, 2009).

Cognitive contributions to visual creativity are likely to differ according to the specific task focus (Nusbaum & Silvia, 2011; Palmiero et al., 2011, 2015). Instructions to generate functional, original, or esthetic ideas may elicit greater evaluation compared to tasks that do not specify the desired nature of generated solutions (Nusbaum & Silvia, 2011). Functional tasks include design tasks in which practical solutions must be generated in response to a specified problem or need. Generating visual solutions to such problems may in turn engage relatively greater manipulation of visual imagery of existing products (Oxman, 2002), inhibition of irrelevant ideas, planning, and evaluation, compared to tasks where solutions are not required to be functional or realistic (Cross, 2001). Emphasizing the originality of generated solutions may engage combination of semantically distant concepts via semantic retrieval (Grabner, Fink, & Neubauer, 2007; Nusbaum & Silvia, 2011).

Prominent existing accounts of the neural basis of creativity include those emphasizing the contribution of increases (e.g., Fink & Benedek, 2014) or, conversely, decreases (Jausovec & Jausovec, 2000) in electroencephalography (EEG) alpha power. Others have proposed a role of right hemispheric dominance in creativity (e.g., Mihov, Denzler, & Förster, 2010), particularly visual creativity (Aziz‐Zadeh et al., 2013; Mendez, 2004; Miller, Boone, Cummings, Read, & Mishkin, 2000; Miller et al., 1998; Seeley et al., 2008; Shamay‐Tsoory, Adler, Aharon‐Peretz, Perry, & Mayseless, 2011). Goel's (2014) related Frontal Lobe Lateralization Hypothesis posits that the right PFC maintains ill‐structured representations that facilitate idea generation in open‐ended visual design problems. Functional neuroimaging evidence for a critical role of alpha power or the right hemisphere in visual creativity has not, however, been formally synthesized.

Meta‐analyses of functional magnetic resonance imaging (fMRI) studies of creativity have reported that not only overlapping regions of bilateral PFC and occipitotemporal cortex contribute to creativity across multiple domains, for example, musical, verbal, and visual (Boccia et al., 2015; Gonen‐Yaacovi et al., 2013), but have also hinted at domain‐specific neural contributions to these types of creativity (Boccia et al., 2015). Given this apparent domain‐specificity and the importance of visual creativity, it is of value to examine the neural basis of visual creativity as distinct from other forms of creativity. Previous reviews have, however, tended to collapse across visual and verbal divergent thinking, or visual art and musical improvization (Dietrich & Kanso, 2010; Gonen‐Yaacovi et al., 2013; Wu et al., 2015).

The present review aimed to establish whether a common neural basis of visual creativity emerges when synthesizing studies examining neural activity associated with this creative modality only, and only studies examining active generation of visual creative ideas (Section 2.1). Unlike previous fMRI meta‐analyses (Boccia et al., 2015; Gonen‐Yaacovi et al., 2013; Wu et al., 2015), studies employing any neuroimaging technique were included.

The neural basis of visual creativity was assessed using activation likelihood estimation (ALE) meta‐analysis of fMRI studies, in addition to qualitative synthesis of findings from this and other neuroimaging modalities. We also evaluated support for existing accounts of cognitive and neural contributions to creativity, including right hemispheric dominance, PFC involvement, and the role of alpha power. A further aim was to expand on previous reviews by assessing evidence for (1) effects of participants’ visual creative ability on the neural or electrophysiological correlates of visual creativity and (2) differences in the neural basis of visual creativity according to whether tasks emphasized the functionality, esthetics, or originality of generated visual solutions (Dietrich & Kanso, 2010; Gonen‐Yaacovi et al., 2013).

## 2 Methods

### 2.1 Search strategy

This systematic review and ALE meta‐analysis followed PRISMA guidelines (Liberati et al., 2009) and synthesized studies recording neural activity during active generation of visual‐based creative (i.e., novel and useful) ideas (Runco & Jaeger, 2012). Tasks involving only passive viewing of visual creative forms or their retrieval from memory were not included. *Convergent thinking*, problem‐solving or insight tasks, which typically have a single, fixed solution, can engage creative thinking (Abraham, 2013). *Divergent thinking* or open‐ended tasks with multiple possible solutions, however, typically provide a closer approximation to the creativity involved in visual design, art, and innovation (e.g., Ellamil et al., 2012; Kowatari et al., 2009), and also meet standard definitions of creativity (Runco & Jaeger, 2012). Consequently, this review focused on functional neuroimaging studies employing open‐ended or divergent thinking visual creativity tasks. Principal searches were conducted from 12 to 18 March 2015 in Web of Science (1864–2015), EMBASE (1947–2015), PsycINFO (1940–2015), PubMed (1950–2015), ScienceDirect (1823–2015), and Compendex (1884–2015). Search terms included “creativity,” “ideation,” “ill‐structured,” “divergent thinking,” “idea generation” (including variants of these terms), co‐occurring with one or more neuroimaging terms: “functional (neuro)imaging,” “PET/positron emission tomography,” “functional magnetic resonance imaging/functional MRI/fMRI,” “electroencephalography/EEG,” “event‐related potential/ERP,” “magnetoencephalography/MEG,” and/or “functional near infrared spectroscopy/FNIR.” Further searches including the terms “electrocorticography/ECoG” and “multiunit activity/MUA” did not yield any additional relevant results. Update searches were conducted in May and June 2015 and March 2016.

**Figure 1:**
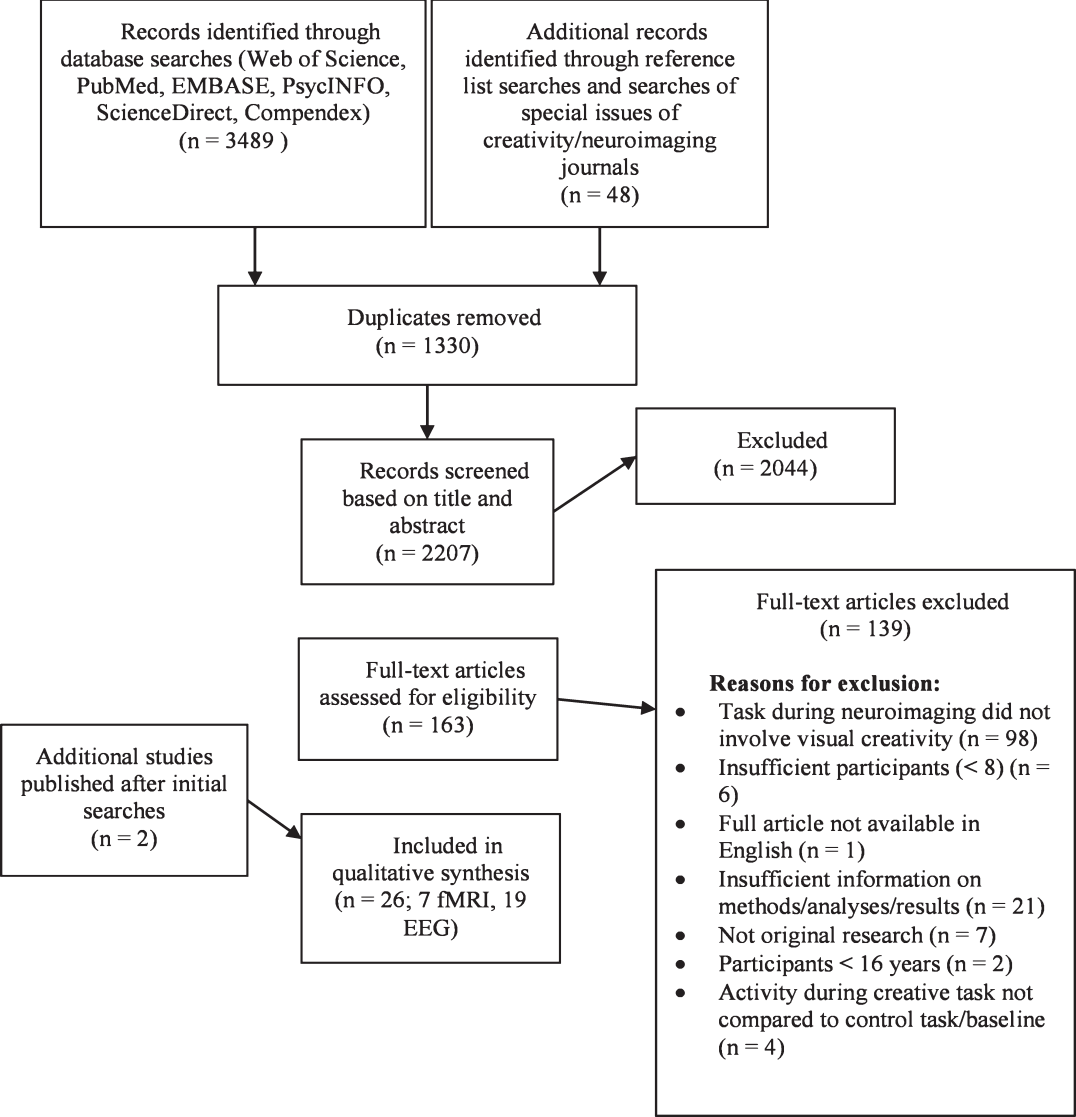
Flowchart of article selection, following PRISMA guidelines. Adapted from Moher et al. (2009). fMRI, functional magnetic resonance imaging; EEG, electroencephalography

The article selection procedure is summarized in Fig. 1. Using the above search terms, 3489 records were identified and 46 were identified through reference lists of relevant studies. Following de‐duplication and screening for inclusion criteria (see Table 1), 26 articles, comprising 27 experiments, were included in the review, of which six fMRI studies were included in the ALE meta‐analysis. No limitations were placed on the date of publication.

**Table 1:** Inclusion criteria

Criterion	
1.	Published in English (translations were accepted)
2.	Peer reviewed
3.	Original research article
4.	Human participants
5.	Include healthy adult participants aged 16 years or above
6.	*N *≥ 8 per experimental group/condition
7.	Use of fMRI, PET, MEG, NIRS, EEG, ERPs, electrocorticography, or multiunit activity to examine neural activity during performance of a task involving visual creativity
8.	Compare neural activity during visual creativity to activity during an appropriate non‐rest control task or to activity during rest/fixation
9.	For fMRI, PET, and NIRS studies, include MNI or Talairach coordinates of peaks of activity for contrasts involving visual creativity
10.	Report details of analyses conducted and significance of results

fMRI, functional magnetic resonance imaging; EEG, electroencephalography.

### 2.2 Quality assessment

Included experiments were rated according to quality assessment criteria adapted from Whiting, Rutjes, Reitsma, Bossuyt, and Kleijnen's (2003) QUADAS quality assessment tool: (1) clear description of participant selection criteria and demographics; (2) visual creativity task compared against a non‐rest/fixation control task (hereafter, “control task”); (3) sufficient detail on task procedure for reproducibility; (4) sufficient detail on the neuroimaging procedure and outcome measures for reproducibility; (5) sufficient information on analyses and results for reproducibility; (6) conclusions justified based on analyses, for example, appropriate multiple comparisons corrections; (7) no substantial confounds between groups/conditions. Criterion (2) was selected as comparisons against a constrained non‐rest control task that elicits overlapping processes are thought to better facilitate isolation of processes that are unique to the task of interest than an unconstrained rest/fixation condition (Abraham, 2014; Lazar, 2008). For each experiment, a score of 0 (criterion not met) or 1 (criterion met) was assigned for each criterion, and the percentage of criteria met was calculated. Scores are summarized in Table S1.

### 2.3 Meta‐analysis strategy

#### 2.3.1 Selection of voxels

Of the seven fMRI studies meeting inclusion criteria (see Fig. 1 and Section 2.1), the six (Aziz‐Zadeh et al., 2013; Ellamil et al., 2012; Gilbert, Zamenopoulos, Alexiou, & Johnson, 2010; Huang et al., 2013; Park, Kirk, & Waldie, 2015; Saggar et al., 2015) which reported 3D coordinates of peaks showing greater activity during visual creativity compared with a non‐rest control task were included in the ALE meta‐analysis. These reported 77 foci in seven contrasts (see Table 2, for tasks), involving 150 participants.

**Table 2:** Summary of reviewed functional magnetic resonance imaging (fMRI) studies

Study	Population	*N*	Mean age (*SD*)	Task	Key findings
Aziz‐Zadeh et al. (2013)	Architects/architecture students	13 (7 F)	23.15 (3.36)	*Creative*: Generate nameable visual object from three presented shapes. Twenty trials. *Control*: Mentally rotate presented parts of shape. 20 trials. Task focus: None	Creative > Control: L SFG (BA 6/8); L IFG (BA 47); L lateral occipital gyrus (BA 39); L MTG (BA 22) Control > Creative: R posterior parietal (BA 40); R postcentral gyrus (BA 3); L postcentral gyrus (BA 2); R precuneus (BA 7); R inferior occipital gyrus (BA 18)
Ellamil et al. (2012)	Art and design students	15 (9 F)	22.14 (2.25)	*Creative*: (a) Generate: design and sketch book covers based on descriptions of documentaries(b) Evaluate: write/sketch evaluations of covers designed in *Generate* stage*Control*: trace linesTask focus: function	Generate > Evaluate: L IFG (BA 45); L cerebellum; bilateral: hippocampus, PHC (BA 36), premotor (BA 6); superior parietal (BA 7), IPL (BA 40), MTG (BA 19), fusiform gyrus (BA 37) Evaluate > Generate: ACC (BA 24/32); precuneus (BA 7); posterior cingulate (BA 23/31); L anterior insula; bilateral: SFG (BA 10); MFG (BA 9); IFG (BA 45, 47); SMA (BA 6); IPL (BA 39/40); superior parietal (BA 7); temporopolar (BA 38); MTG (BA 22); cuneus (BA 19); MOG (BA 18); lingual gyrus (BA 17); cerebellumPositive correlation self‐rated generation success and Generate > Evaluate activity in: bilateral PHC, IPL and premotor area. Positive correlation between self‐rated evaluation and Evaluate > Generate activity in: ACC, bilateral premotor area; LIFG; superior parietal lobe; fusiform; MTG; L cerebellum
Gilbert et al. (2010)	Adults with design experience	18 (11 F)	37	*Creative (Design)*: Ill‐structured design—design room layout to meet brief. (a) *Study*: read instructions, plan solution; (b) *Perform*: implement solution by moving furniture on screen*Control (Problem solving)*: Well‐structured problem solving—arrange room layout. *Study* and *perform* phases as above. Task focus: Function	Across design and problem solving; Study > Perform: L and R vmPFC (BA 11); R DLPFC (BA 9, 46); R premotor (BA 6); R lateral temporal (BA 21); R lateral parietal (BA 40); R medial occipital cortex (BA 18). In R DLPFC ROI, greater activity for design versus problem solving during the study phase. Within regions showing Perform > Study activity, right thalamus showed greater activity during design versus problem solving.
Huang et al. (2013)	Healthy adults	26	22 (1)	*Creative*: TTCT‐IF(a) Generate novel and interesting image (IN1); (b) Generate image, no emphasis on novelty (IN2) Task focus: Originality/fluency	IN2 > baseline: Bilateral postcentral gyri (BA 2/3); superior parietal lobule (BA 5/7); calcarine (BA 17, 18), lingual (BA 19), and fusiform gyri (BA 37); IPL (BA 39/40); IFG (BA 44/45/47); MFG (BA 9/46); hippocampus; insula (BA 13); precentral gyri (BA 6) L SFG (BA 6/8/10) IN1 > IN2 (small volume corrected): L MFG (BA 9); L IFG (BA 11/46/47); L precentral gyrus; R MOG (BA 18) IN2 > IN1: R MFG (BA 10/46); L IPL (BA 6) ROI analysis: IN1 > IN2 in L mPFC (BA 9); IN2 > IN1 in R mPFC (BA 9)
Kowatari et al. (2009)	“*Experts*”: Art and design students “*Novices*” (non‐Art and design students)	Experts: 20, 12 FNovices: 20, 12 F	Range: 20–28	*Creative (Design)*: Generate pen designs while looking at photos of pens*Control*: count number of pens presentedTask focus: function	Whole‐brain: no differences between experts and novices in design or control activity (vs. baseline); no differences between design and control tasks in experts or novices. ROIs in PFC and parietal cortex: R > L in experts but not novices. In experts, R versus L difference in PFC positively associated with originality of pen designs.
Park et al. (2015)	Healthy adults. High and low schizotypy (HS, LS)	48 (31 F)	23.42 (4.50)	*Creative*: TTCT‐IF. 10 trials. *Control*: Trace dotted line. 10 trials. *Baseline*: fixate on paperTask focus: Originality/fluency	Greater task‐related activation for Creative versus Control: L MFG (BA 6^a^); L ITG (BA 37^a^); R ITG (BA 20/37^a^); R angular gyrus (BA 7/19^a^) Reduced task‐related deactivation in creative versus control: L superior medial frontal gyrus (BA 6/8^a^); L insula (BA 13^a^); R IFG (BA 47^a^); R MOG (BA 19^a^); L IPL (BA 7^a^); L thalamus; R PHG (BA 19^a^)
Saggar et al. (2015)	Healthy adults	30 (16 F)	28.77 (5.54)	*Creative*: Draw visual representation of a given word on drawing tablet*Control*: Draw zigzag lines on drawing tabletTask focus: Function	Creative > Control: Bilateral paracingulate gyrus (BA 32); L MFG/SFG (BA 6); bilateral cerebellum; L LOC (BA 19); L superior parietal lobule (BA 7); L precuneus (BA 7); R MFG/SFG (BA 6); R IFG (BA 13/45) Control > Creative: R STG (BA 22/38/41); R medial frontal gyrus (BA 11); L parietal (BA 39); L MTG; L STGNegative correlation quality of drawings and activity in paracingulate gyrus cluster. Positive correlation increased subjective task difficulty and activity in: L MFG/precentral gyrus (BA 6/9/8); L IFG (BA 45). Increased activity associated with increased creativity ratings in: cerebellum; brain stem

F, female; TTCT‐IF, Torrance Test of Creative Thinking—Incomplete Figures; L, left; R, right; ACC, anterior cingulate cortex; BA, Brodmann area; DLPFC, dorsolateral prefrontal cortex; IFG, inferior frontal gyrus; ITG, inferior temporal gyrus; IPL, inferior parietal lobule; LOC, lateral occipital cortex; MFG, middle frontal gyrus; MOG, middle occipital gyrus; mPFC, middle prefrontal cortex; MTG, middle temporal gyrus; PHC, parahippocampal cortex; PHG, parahippocampal gyrus; SFG, superior frontal gyrus; SMA, supplementary motor area; vmPFC, ventromedial PFC.

Unless otherwise stated, “baseline” refers to fixation.

a BA approximate, as not reported by Park et al. (2015) but estimated by LMP based on coordinates using Talairach Daemon.

#### 2.3.2 Activation likelihood estimation

A coordinate‐based ALE meta‐analysis was conducted using Brainmap GingerALE 2.3 (http://www.brainmap.org/ale). ALE meta‐analysis uses peak coordinates from published studies to calculate brain regions in which the convergence across studies is greater than expected by chance if the included foci were independently distributed (Eickhoff, Bzdok, Laird, Kurth, & Fox, 2012; Eickhoff et al., 2009). Each included activation focus is modeled as the center of a 3D Gaussian distribution, the full width at half maximum of which is determined by the study's sample size (Eickhoff et al., 2009). Modeled activation (MA) maps are calculated by computing the maximum across the Gaussian distribution of each focus (Turkeltaub et al., 2012). The ALE map resulting from combining the MA maps is then compared against an ALE null distribution map. A random effects model was employed (Eickhoff et al., 2009), and significance thresholds on the ALE scores were set via cluster‐level inference (Eickhoff et al., 2012). A cluster‐level threshold of *p *<* *.05 and cluster‐forming threshold of *p *<* *.001 were used to set the minimum cluster volume at 192 mm^3^, via 1000 permutations. The smaller, more conservative mask size was selected.

The meta‐analysis was conducted in MNI space. In GingerALE 2.3, anatomical labels were assigned to ALE peaks which surpassed the voxel and cluster‐level thresholds using the Talairach Daemon, after transformation of significant coordinates using icbm2tal (Lancaster et al., 2007).

## 3 Results

Included studies comprised 7 fMRI and 19 EEG experiments. No NIRS, MEG, ERP, or PET studies met inclusion criteria. Information on participants, creative and control tasks, analyses, and results are summarized in Table 2 for fMRI studies, and Table 4 for EEG studies.

### 3.1 Study characteristics

Of the 26 reviewed articles, 10 have to our knowledge not been included in previous systematic reviews or meta‐analyses. The 26 articles comprised 27 experiments and around 800 participants—this is approximate as the studies of Bechtereva and Nagornova (2007) and Nagornova (2007), of Petsche (1996) and Petsche, Kaplan, Von Stein, and Filz (1997), and of Volf, Tarasova, and Razumnikova (2010a) and Volf and Tarasova (2010) were conducted using overlapping samples, without stating numbers of participants included in both. Mean sample size was 27 (*SD *= 13) for fMRI studies, and 38 (*SD *= 13) for EEG studies. Participants were aged around 17–60 years (approximate as age not always reported). The most common visual creativity task was the Torrance Test of Creative Thinking—Incomplete Figures (TTCT‐IF; Torrance, 1974) or variants of this task, used in 11/27 (41%) experiments. In this task, part of Torrance's (1974) standardized battery of verbal and nonverbal creative thinking tasks, participants mentally generate a complete image from a presented fragment of a drawing. Measures of fluency (number of ideas) and originality (statistical infrequency of ideas) are typically recorded. The next most common task, employed in six (22%) experiments, involved generating images by mentally combining presented shapes. Studies differed in whether idea generation and externalization (via sketching/verbalization) occurred in the same (e.g., Park et al., 2015; Saggar et al., 2015) or distinct (e.g., Volf et al., 2010a) phases. Tasks included those in which solutions must fulfill a specified function (25.9%), those emphasizing the originality/fluency of solutions (51.9%; these are combined as typically task instructions emphasized both criteria, for example, “generate as many original solutions as possible”), those emphasizing the esthetics of solutions (7.4%), and tasks giving no instructions as to the desired characteristics of solutions (14.8%). On average, studies satisfied 67% of quality criteria (*SD *= 21; range 14–100%; Table S1). Quality scores did not differ between fMRI (*M *= 76%, *SD *= 14) and EEG (*M *= 64%, *SD *= 23) studies (*t*(25) * *= 1.21, *p *=* *.24).

### 3.2 Functional magnetic resonance imaging studies

#### 3.2.1 Functional magnetic resonance imaging study characteristics

The participants, procedure, and main findings of the reviewed fMRI studies are summarized in Table 2. Two studies employed the TTCT‐IF, with instructions emphasizing the originality of generated solutions, although Huang et al. (2013) compared activity during efforts to generate unique solutions against activity during generation of any appropriate solutions; while Park et al. (2015) compared activity during simultaneous generation and sketching of solutions against activity during line tracing. Four studies employed tasks emphasizing a specific function of generated solutions. Of these, three were visual design tasks—designing and sketching book covers (Ellamil et al., 2012), generating pen designs (Kowatari et al., 2009), and an ill‐defined room layout task (Gilbert et al., 2010). In the study by Saggar et al. (2015), participants were asked to draw visual representations of presented words (e.g., “graduate,” “snore”).

In the final study (Aziz‐Zadeh et al., 2013), the desired features of visual solutions were not emphasized, and brain activity was recorded from architects while they mentally combined three presented shapes to create an image. Activity during this task was compared with activity during a mental rotation task.

#### 3.2.2 ALE meta‐analysis findings

**Figure 2:**
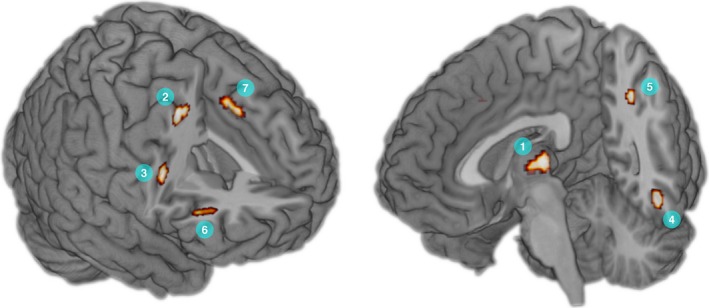
Thresholded ALE map (cluster‐level threshold *p* < .05, cluster‐forming threshold *p *< .001, uncorrected at the voxel level), showing significant clusters for the contrast of visual creativity versus non‐rest control tasks. Results are illustrated using the “ch256” template supplied with MRIcroGL software (http://www.mccauslandcenter.sc.edu/mricrogl/). Cluster numbers correspond to those listed in Table 3: (1) mediodorsal thalamic nucleus; (2) right middle frontal gyrus; (3) right precentral gyrus; (4) left fusiform gyrus; (5) left angular gyrus; (6) right inferior frontal gyrus; (7) left cingulate gyrus. See Table 3 for MNI coordinates of maxima, cluster sizes, and corresponding ALE values

Six fMRI studies (Aziz‐Zadeh et al., 2013; Ellamil et al., 2012; Gilbert et al., 2010; Huang et al., 2013; Park et al., 2015; Saggar et al., 2015), including 77 foci from seven contrasts were included in the ALE meta‐analysis (Section 2.3). All foci were associated with greater activity during visual creativity compared to control conditions. An additional study (Kowatari et al., 2009) met inclusion criteria but as no differences were found between visual creativity and control tasks in experienced or novice designers, no foci were included in the meta‐analysis. Three included studies employed tasks emphasizing the function of solutions, two emphasized originality/fluency, and one had no clear focus—the numbers of studies in each of these categories were insufficient for analysis of effects of task focus. The meta‐analysis revealed seven clusters that surpassed the significance threshold (see Section 2.3, for thresholding and analysis). Results are summarized in Table 3 and significant clusters are displayed in Fig. 2. Regions showing significant ALE activity included thalamocortical nucleus, right middle and inferior frontal gyri, cingulate gyrus, and left fusiform gyrus.

**Table 3:** Clusters showing significant activation likelihood estimate (ALE) values for the contrast of visual creativity > non‐rest control tasks

Cluster number	Anatomical label	Brodmann Area	Peak MNI coordinates			Cluster size (mm^3^)	ALE value
			*x*	*y*	*z*		
1	Mediodorsal thalamic nucleus	–	0	*−*20	6	648	0.0165
*1*	*Thalamus*	–	*0*	*−12*	*4*	–	*0.0103*
2	Right middle frontal gyrus	6	28	4	50	624	0.0152
*2*	*Right middle frontal gyrus*	*6*	*32*	*−2*	*58*	–	*0.0101*
*2*	*Right cingulate gyrus*	*24*	*20*	*4*	*50*	–	*0.0100*
3	Right precentral gyrus	6	44	6	24	488	0.0165
4	Left fusiform gyrus	37	*−*48	*−*54	*−*10	376	0.0138
5	Left angular gyrus	39	*−*26	*−*54	40	272	0.0108
*5*	*Left parietal lobe*	–	*−28*	*−50*	*40*	–	*0.0103*
6	Right inferior frontal gyrus	13	40	32	6	224	0.0105
*6*	*Right inferior frontal gyrus*	*45*	*46*	*26*	*8*	–	*0.0098*
7	Left cingulate gyrus	32	*−*2	22	42	216	0.0104
*7*	*Left medial frontal gyrus*	*32*	*−4*	*14*	*46*	–	*0.0098*

MNI, Montreal Neurological Institute.

Values associated with subpeaks are displayed in italics.

For each significant ALE cluster, only two studies from a subset of three (Ellamil et al., 2012; Park et al., 2015; Saggar et al., 2015) contributed foci which fell within the cluster boundaries. This meets the previously suggested quality criterion of a contribution of 33% of included studies for reporting ALE maxima (Brooks et al., 2012; Van der Laan, De Ridder, Viergever, & Smeets, 2011), and further foci from other studies which were out with the cluster boundaries may still have contributed to their significance (Brooks et al., 2012). However, due to the possibility that only a minority of studies contributed to the meta‐analysis findings, a qualitative synthesis of fMRI findings is reported below.

#### 3.2.3 Qualitative synthesis

Evidence of greater occipitotemporal engagement during visual creativity compared to control tasks was reported in five of the seven fMRI studies, with activation peaks observed in right middle occipital gyrus (MOG) (Ellamil et al., 2012; Huang et al., 2013; Park et al., 2015), left MOG (Ellamil et al., 2012), bilateral inferior temporal gyri (Park et al., 2015), and left lateral occipital cortex (Aziz‐Zadeh et al., 2013; Saggar et al., 2015).

Two studies reported greater medial temporal lobe activity during visual creativity compared to control tasks. Ellamil et al. (2012) reported greater hippocampal and parahippocampal activity during generation compared to evaluation of visual book cover designs, and Park et al. (2015) reported greater right parahippocampal activity during generation and sketching of TTCT‐IF solutions compared to line tracing. Studies that involved simultaneous sketching and idea generation reported recruitment of left insular cortex, bilateral cerebellum, and thalamus (Park et al., 2015; Saggar et al., 2015).

Regions of left PFC, including superior frontal gyrus (SFG), inferior frontal gyrus (IFG), (Aziz‐Zadeh et al., 2013), middle frontal gyrus (MFG) (Park et al., 2015; Saggar et al., 2015), and premotor cortex (Aziz‐Zadeh et al., 2013; Ellamil et al., 2012) were reported in five of seven studies to show greater activity during visual creativity compared to control tasks, and Huang et al. (2013) reported that left MFG and IFG were more active during generation of original compared to standard responses. Saggar et al. (2015) found that left MFG and IFG activity increased along with increases in subjective ratings of task difficulty, while activity in a left dorsolateral PFC (DLPFC) cluster was positively associated with independent ratings of how well drawings met task demands.

Fewer studies reported right PFC (3/7) compared to left PFC engagement (5/7), but the meta‐analysis findings are indicative of greater between‐study consistency in the localization of right PFC regions. Two studies assessed the lateralization of PFC contributions to visual creativity. Kowatari et al. (2009) reported greater activity during pen design in right compared to left PFC and parietal regions of interest (ROIs) (subregions and Brodmann areas not reported) in art and design students (“experts”), but not in novices. The extent of right over left PFC dominance correlated with ratings of the originality of pen designs, interpreted as facilitation of visual creativity in experts via heightened right PFC activity. However, as Kowatari et al. (2009) in fact observed no significant differences in activity at the whole‐brain level between the design and control task, nor between experts and novices, their findings do not speak directly to the activity supporting visual creativity—the greater right PFC activity in experts may be a global effect which is not specific to visual creativity. Furthermore, the authors did not test the Hemisphere × Group interaction which would be necessary to support right hemispheric dominance in experts only.

Huang et al. (2013) reported greater activity in left compared to right medial PFC (mPFC) ROIs under instructions to produce original solutions, while the opposite pattern emerged while producing standard solutions. A left over right inhibitory mechanism was proposed, but again the relevant Hemisphere × Task interaction was not assessed.

### 3.3 Electroencephalography studies

#### 3.3.1 Electroencephalography study characteristics

Nineteen EEG studies comprising 20 experiments were reviewed. The main findings are summarized in Table 4. Most experiments (60%) employed tasks emphasizing the originality and/or fluency of solutions, including the TTCT‐IF or similar variants, or mental combination of shapes to form original images. In two studies (Bhattacharya & Petsche, 2005; Petsche et al., 1997, Experiment 1), participants generated esthetically pleasing images, while in three studies, participants generated functional solutions, for example, generating a novel visual intelligence test (Jaarsveld et al., 2015) or a visual representation of an abstract concept (Petsche, 1996; Petsche et al., 1997, Experiment 3).

**Table 4:** Summary of reviewed electroencephalography (EEG) studies. Unless otherwise stated, “baseline” refers to fixation

Study	Population	N	Mean age (*SD*)	Task	Outcome measures	Key findings
Bechtereva and Nagornova (2007)	Healthy adults	30	20	*Creative*Cr1: draw original picture using simple shapesCr2: draw in an original manner a designated object using simple shapes*Control*: C1: draw from memory a previously presented pictureC2: Continuously draw simple shapesTask focus: Originality/fluency	Coherence	*Creative* versus *control*: Increased theta coherence between frontal and parietal sites; increased alpha1 and alpha2 coherence, left frontal and temporal foci; decreased interhemispheric beta2 and gamma coherence; increased intrahemispheric beta2 and gamma coherenceCreative versus baseline: increased delta and theta coherence; decreased alpha, beta, and gamma coherence
Bhattacharya and Petsche (2005)	Female artists and novices	19 (19 F) Artists: 9Novices: 10	Artists: 44.3Novices: 37.5	*Creative*: mentally generate drawing while fixating on wallTask focus: Esthetics	Synchronization (coherence)	*Creative* versus *fixation*: Artists: delta synchronization, including frontoposterior. Greater delta synchronization in artists versus novices, particularly occipitotemporal sites. Novices: beta and gamma synchronization over frontal sites. Greater alpha, beta, and gamma synchronization in novices versus artists, particularly at frontal sites. Artists and novices: greater RH versus LH synchronization.
Jaarsveld et al. (2015)	Healthy adults	52 (31 F)	24.33 (4.25)	*Creative*: Creative Reasoning Task (CRT): Toolbox—generate images. Rows 1–3: generate visual problem‐solving task using images from Toolbox phase. Generate and drawing phases for each. 10 trials. *Control*: successive rows of matrix involve more CT. Task focus: function	Task‐Related Power (TRP)	Greater alpha TRD in Toolbox phase (DT) compared to alpha synchronization in later stages (CT); particularly over frontal and posterior sites. Increased alpha synchronization at frontal sites at first and last time intervals.
Jausovec and Jausovec (2000) (Experiment 2)	Healthy adults	30 (18 F)	Range: 18–19	*Creative: Dialectic*: (a) read text; b) generate essay*DT*: c) verbal—generate things that make noise; Alternative Uses Task; generate similarities between radio and phone d) visual—TTCT‐IF (3 trials) *Baseline*: fixate screen while listening to musicTask focus: Originality/fluency	Power; coherence	*Power*: Reduced alpha power for visual DT versus verbal DT and dialectic tasks, restricted to occipital and left frontal sites for alpha2. *Coherence*: Visual DT versus dialectic: reduced alpha1 coherence during between right frontal and right parietal sites, and between left frontal and temporal sites. Reduced alpha2 coherence between right frontal and bilateral parietal sites. Increased frontal alpha2 coherence. Visual and verbal DT versus dialectic: Reduced alpha2 coherence.
Jausovec (2000) (Experiment 2)	Healthy adults. High and average IQ (HIQ, AIQ); high and average creativity (HC, AC) groups	48	–	As above (Jausovec and Jausovec 2000)	Power; coherence	*Power*: HC participants showed greater alpha1 and alpha2 power versus AC during creative tasks (verbal and visual DT; dialectic). HC and HIQ showed greater right frontal alpha1 power versus AC and AIQ. *Coherence*: HC: greater inter‐ and intrahemispheric alpha1 coherence versus AC during all tasks. Reduced alpha2 coherence versus AC during verbal and visual DT. HIQ: reduced alpha1 coherence versus AIQ. Greater alpha2 coherence versus AIQ.
Kozhedub et al. (2007)	Graphic Arts students	23	Range 20–30	*Creative*: Generate visual images from simple elements (right angles, diagonal lines). Images sketched after generation. Produced images classified based on independent ratings as “standard” or “original”Task focus: Originality/fluency	Coherence (number of coherence links)	*Creative* versus *baseline*: Increased interhemispheric beta2 coherence links; increased inter‐ and intrahemispheric delta links; decreased alpha and beta1 links. Greater alpha2 coherence decreases in LH versus RH. Changes in coherence versus baseline positively correlated between right frontal and right parietal sites. *Generation of original* versus *standard images*: reduced number of alpha2 coherence decreases versus baseline.
Molle et al. (1999)	Healthy adult males, high and low DT performance groups (HDT, LDT)	28 (0 F)	26	*Creative*: Verbal DT: (a) DivVerb1—Consequences task; (b) DivVerb2—Alternative Uses TaskVisual DT: (c) DivVis1—generate funny similarities between images; (d) DivVis2—Variant of TTCT‐IF *Control*: Verbal CT: (e) ConVerb1 and (f) ConVerb2—textual problem solvingVisual CT: (g) ConVis1, (h) ConVis2—find correct continuation of letter seriesTask focus: Originality/fluency	Dimensional complexity (DC); power	*DC*: Greater DC for DT (verbal and visual) versus CT. Reduced DC for HDT versus LDT at central and parietal sites for DivVis1, and at frontal sites for DivVist2. *Power*: Reduced delta and theta power for DT versus CT; reduced alpha power for DT versus baseline. Greater beta power for DT versus CT at central and posterior sites; greater beta power for DT versus baseline at posterior sites. Across all DT tasks, greater beta power for LDT versus HDT over frontal sites.
Nagornova (2007)	Healthy adults (same sample as Bechtereva and Nagornova, 2007; above)	30	20	Same as Bechtereva and Nagornova (2007), above	Power	*Creative* versus *control*: Increased beta2 and gamma power; some increases in alpha power; reduced beta1 power*Creative* versus *baseline*: Predominant reductions in alpha power and increases in beta and gamma power. At frontotemporal sites, drawing original pictures (Cr1) associated with lower beta1 and gamma power than drawing a specified object in an original manner (Cr2).
Petsche et al. (1997) (Experiment 1)	Healthy adult females, half educated in Fine Arts	38 (38 F)	–	*Creative*: Generate original painting. Painting sketched after EEG recording. *Control*: (a) View painting; (b) Memorize painting; (c) Read textTask focus: esthetics	Power; coherence (alpha1, alpha2)	*Power*: Decreased alpha power for viewing, memorizing, and mentally generating pictures versus baseline. Alpha power reductions smaller for artists versus novices for Creative and Control tasks. *Coherence*: versus baseline, overall increased coherence ‐long‐range inter‐ and intrahemispheric increases, particularly at posterior sites.
Petsche et al. (1997) (Experiment 3)	Healthy adults	38 (18 F)	–	*Creative*: Generate an image to represent an abstract concept. Sketch images after EEG. Task focus: Function	Coherence (alpha1, alpha2)	*Creative* versus *baseline*: Males: long‐range interhemispheric alpha1 coherence decreases; short‐range interhemispheric posterior alpha2 coherence increasesFemales: alpha 1 inter‐ and intrahemispheric coherence decreases at frontal sites; left frontoparietal‐ right frontal increases in alpha2 coherence
Petsche (1996) (Experiment 2)	Healthy adult females. Half educated in Fine Arts	38 (all F)	–	Same as Petsche et al. (1997), above	Coherence (task vs. baseline)	*Creative* versus *baseline*: Many long‐range intra‐ and interhemispheric coherence increases in all frequency bands, most pronounced in theta, alpha1 and beta2. Some decreases in interhemispheric frontal coherence in delta, theta, alpha1, alpha2 and beta1 bands.
Razumnikova et al. (2009)	Healthy adults	53 (26 F) Verbal task: 27Visual task: 26	–	*Creative*: Visual: TTCT‐IFVerbal: Generate sentence from word triadsInstructions 1 (IN1): create figure/sentenceInstructions 2 (IN2): create original figure/sentenceTask focus: Originality/fluency	Power; coherence	*Power*: Creative (visual and verbal) versus baseline: reduced alpha; increased beta2 power for IN1 but not IN2. Theta1 power decreased for visual versus baseline; increased for verbal versus baseline. *Coherence: Visual creative* versus *baseline*: increased theta and beta2 coherence—beta2 increases particularly evident in men. Women showed greater RH alpha2 coherence and reduced RH beta2 coherence for IN2 versus IN1.
Razumnikova et al. (2010)	Healthy adults	65 (34 F) Verbal task: 39 (21 F) Visual task: 26 (13 F)	18.4 (1.1)	*Creative*: Verbal: Remote Associates Task—think of associate of three presented words, give most original response. 10 trials. Visual: TTCT‐IF. 10 trials. Task focus: Originality/fluency	Beta2 power; coherence	*Power*: Increased beta2 power for creative (visual and verbal) versus baseline; increase greater for verbal versus visual. *Coherence*: Increased inter‐ and intrahemispheric beta2 coherence for creative versus baseline over many sites; decreased interhemispheric frontal coherence. Greater task‐related increases in coherence for verbal versus visual at frontal sites. Men: greater task‐related frontal interhemispheric coherence increases versus women. Women: originality negatively correlated with task versus baseline coherence differences
Sviderskaya et al. (2006)	Graphic Art students (artists); nonart students (novices).	Artists: 23 (19 F) Novices: 39 (15 F)	Artists: 26.51 (3.67) Novices: 31.00 (4.51)	*Creative*: Generate images from varying numbers of presented simple elements (angles, lines). Images then sketched and classified via independent ratings as “standard”/“original” Task focus: None	Spatial synchronization (SS); spatial disordering (SD); coherence; power	*SS* versus *SD*: Artists showed greater increases and decreases in SS and SD versus novices. Greater increases in SS and SD in artists versus novices at right anterior sites; greater increases in novices versus artists at right occipital sites. With increasing numbers of elements, artists showed right anterior and left temporal increases in SS and SD. Novices showed increased left anterior and right occipital SS. *Coherence and power*: Generating images from >8 elements, artists showed greater theta, delta, alpha and beta coherence and power versus novices
Sviderskaya (2011a)	Graphic Art students (artists); nonart students (novices). Subsample from Sviderskaya et al., 2006	Artists: 23 (19 F) Novices: 34 (14 F)	Artists: 26.51 (3.67) Novices: 31.23 (4.51)	*Creative*: Generate images from simple elements. Based on sketches after EEG, trials classified as successful/unsuccessful. Task focus: None	Spatial synchronization (SS); spatial disordering (SD); coherence; power; informational energy	*SS and SD*: Successful performance versus baseline: Artists: increased right frontotemporal and left parieto‐occipital SS and SD. Novices: increased left frontotemporal and right parieto‐occipital SS and SD versus baseline. Unsuccessful performance versus baseline: Artists showed increased SD in all regions. *Coherence*: Successful performance versus baseline: Artists: increased delta, alpha1 and beta coherence, left occipital and right frontal foci. Novices: increased L frontal alpha coherence. *Power*: Successful versus baseline: Both groups: reduced delta, theta and alpha power; increased beta1 and beta2 power*Informational energy*: Artists: greater informational energy versus novices for upper alpha—upper beta bands
Sviderskaya (2011b)	Healthy adult males	30 (0 F)	Range: 35–50	*Creative*: Visual DT (VisDT): generate images by combining simple shapesVerbal DT (VerbDT): generate as many words as possible from 2 letters*Control*: Visual CT (VisCT): Determine which image a fragment belongs toVerbal CT (VerbCT): complete gaps in word listTask focus: None	Spatial synchronization (SS); coherence	*VisDT* versus *VisCT*: Greater right anterior and left posterior SS, for delta, alpha and beta bands. *VisDT* versus *VerbDT*: Greater R anterior temporal SS, in beta range (22.5–24 Hz) *VerbDT* versus *VisDT*: Greater SS in L anterior regions, in alpha1 (8.25–11 Hz)
Volf and Tarasova (2010)	Healthy adults. High and low‐creativity groups (HC, LC) based on originality of generated figures.	28 (14 F)	Range: 18–21	*Creative*: TTCT‐IFIN1: Instructed to generate imagesIN2: Instructed to generate original imagesTask focus: Originality/fluency	Power; task‐related synchronization (TRS); task‐related desynchronization (TRD)	*Creative* versus *baseline*: Reduced theta and increased beta power*IN1* versus *baseline*: HC men showed beta1 TRD, HC women showed beta1 TRS. HC women showed greater beta1 power in posterior versus frontal regions.
Volf and Tarasova (2014)	Healthy adults	31 (16 F)	Range: 18–21	*Creative*: TTCT‐IF. IN1: Generate original images. 2 trials. IN2: Participants informed they would receive a monetary reward for generation of original images. 2 trials. Task focus: Originality/fluency	Power (theta, alpha, beta)	*Creative* versus *baseline*: Reduced alpha power*IN2* versus *IN1*: Reduced theta power at baseline and during task. Decreased beta power over posterior sites during task. Increased alpha power at baseline.
Volf et al. (2010a)	Healthy adults. High and low‐creativity groups (HC, LC) based on originality of generated figures.	28 (14 F)	Range: 18–21	*Creative*: TTCT‐IFIN1: Generate image from incomplete figureIN2: As above, and instructed to generate original imageTask focus: Originality/fluency	Task‐related power (TRP; log transform of power during task—power at baseline)	*Creative* versus *baseline*: Reduced alpha power*IN1* versus *baseline*: In parietotemporal regions, HC men showed greater alpha TRP reductions versus LC men. For alpha1, HC men showed greater TRP reductions in posterior versus anterior sites. For alpha2, all groups except LC men showed greater TRP reductions in posterior versus anterior sites. For alpha2, greater reductions in TRP for HC men versus LC men; but greater TRP reductions for LC women versus HC women. *IN2* versus *baseline*: Reduced TRP over posterior sites, effect stronger in RH. LC men showed reduced TRP versus HC men in RH.
Volf et al. (2010b)	Healthy adults—HC and LC groups based on originality of generated figures	40 (20 F)	Range: 18–21	*Creative*: TTCT‐IFTask focus: Originality/fluency	Coherence	*Creative* versus *baseline*: LC: decreased theta2, alpha1 and alpha2 coherence. HC: increased theta2 and alpha1 power. HC showed smaller alpha2 decreases versus LC. LC men: reduced alpha2 intrahemispheric coherence in left anterior and right posterior regions. HC men: reduced task‐related decreases in alpha2 coherence versus LC men.

RH, right hemisphere; LH, left hemisphere; alpha1, lower alpha (~8–10 Hz); alpha2, upper alpha (~ 10–14 Hz); beta1, lower beta (~12.5–16 Hz); beta2, upper beta (~ 16.5–30 Hz); DT, divergent thinking; CT, convergent thinking; TTCT‐IF, Torrance Test of Creative Thinking—Incomplete Figures.

Most experiments employed measures of EEG power (25%), coherence (35%), or both (35%). EEG power refers to the amplitude of a particular frequency band, while coherence, or phase synchrony, instead reflects functional cooperation between cortical regions. These measures were most often recorded for the lower (~8–10 Hz) and upper (~10–14 Hz) alpha bands (e.g., Jausovec, 2000; Petsche et al., 1997). Several studies reported effects in the delta (<4 Hz), theta (4–7 Hz), beta (14–31 Hz), and gamma bands (>31 Hz).

Studies varied substantially in the control tasks employed, and the focus of key contrasts. Several compared activity during visual creativity to a verbal creativity or verbal and/or visual control task, often involving memory or convergent thinking (e.g., Jausovec, 2000; Nagornova, 2007). Many, however, simply compared electrophysiological activity during visual creativity against a baseline fixation/rest condition (e.g., Bhattacharya & Petsche, 2005; Jausovec & Jausovec, 2000; Kozhedub, Sviderskaya, & Taratynova, 2007). Eight studies compared activity between individuals of high and low creativity and six compared generation of original versus standard creative images.

There were insufficient experiments employing functional or esthetic task foci for direct comparison with studies emphasizing originality/fluency of solutions, but no clear differences in qualitative findings emerged when examining tasks focusing on originality/fluency separately from other studies. The summary of findings below therefore combines across task foci, and is organized according to outcome measures (power, coherence; other), and the main contrasts employed: (1) visual creativity versus baseline rest/fixation; (2) visual creativity versus non‐rest control task(s); (3) individuals of high versus low creativity; (4) generation of original versus standard visual images.

#### 3.3.2 Findings—Electroencephalography power

**Figure 3:**
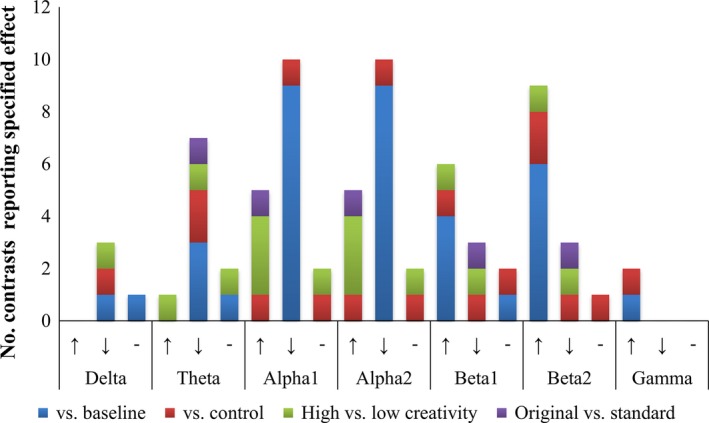
Summary of the frequency (number of contrasts showing relevant effect) with which studies reported predominant increases (↑), predominant decreases (↓), and no clear pattern of increases or decreases (−) in power in each frequency band. Findings of power changes during visual creativity versus baseline are displayed in blue; power changes versus control tasks in red; differences between high‐ and low‐creativity participants in green; and differences between production of original versus standard images in purple

Figure 3 summarizes the numbers of studies where a substantial majority of significant effects on EEG power across electrodes were (1) increases, (2) decreases (hereafter, “predominant power increases” and “predominant power decreases,” respectively), and (3) where null effects or no clear pattern of power increases or decreases emerged. These outcomes are summarized for each of the main contrast types (Section 3.3.1).

##### 3.3.2.1 Visual creativity versus baseline

In the low‐frequency delta and theta bands and the lower and upper alpha bands, a consistent pattern of decreases in EEG power during visual creativity compared to baseline fixation or rest—*task‐related desynchronization* (TRD) emerged across studies (Jaarsveld et al., 2015; Jausovec & Jausovec, 2000; Petsche et al., 1997; Volf & Tarasova, 2010, 2014; Volf et al., 2010a). Predominant increases in power versus baseline, *task‐related synchronization* (TRS), were observed in the high‐frequency beta and gamma bands (Molle, Marshall, Wolf, Fehm, & Born, 1999; Nagornova, 2007; Razumnikova, Volf, & Tarasova, 2009, 2010; Sviderskaya, 2011a; Volf & Tarasova, 2010). These TRD and TRS effects were typically widespread over multiple electrode sites, including bilateral frontal, central, and occipital sites. Two studies, however, reported that alpha (Volf et al., 2010a) and theta (Volf & Tarasova, 2010) TRD during visual creativity versus baseline was of greater magnitude at posterior compared to anterior sites. Consistent with greater posterior effects, Molle et al. (1999) reported task‐related increases in beta power at parieto‐occipital sites only.

##### 3.3.2.2 Visual creativity versus control

Several studies compared activity during visual creativity to that during verbal creativity (Jausovec & Jausovec, 2000; Razumnikova et al., 2009, 2010), or during verbal and/or visual convergent problem‐solving or memory tasks (Molle et al., 1999; Nagornova, 2007). Molle et al. (1999) observed reduced delta and theta power for divergent compared to convergent thinking tasks, as well as increased upper beta power over central parietal sites, these effects did not differ according to the modality (visual, verbal) of divergent and convergent tasks. Other findings point to greater task‐related power reductions in the theta (Razumnikova et al., 2009) and lower and upper alpha bands (Jausovec & Jausovec, 2000) for visual compared to verbal creative tasks. The latter reductions in upper alpha power were observed over occipital and left frontal sites only.

Consistent with Molle et al.'s (1999) findings of increased upper beta power during visual and verbal creativity, Nagornova (2007) observed greater power in the upper beta band, and in the lower and upper alpha and gamma bands when comparing visual creativity to drawing figures from memory. Razumnikova et al. (2010) also reported creativity‐related upper beta power increases, but these increases were reduced in magnitude for visual compared to verbal tasks. Molle et al. (1999) and Razumnikova et al. (2009) did not reveal significant power differences between visual creative and control tasks in the alpha and beta bands, respectively (Fig. 3).

Two studies compared generation of original and standard solutions to visual creative tasks. Volf and Tarasova (2014) found that under conditions of reward for producing original solutions, baseline and task‐related theta power were reduced and baseline alpha power increased, compared to conditions of no reward. That these effects observed at baseline and during task performance are consistent with a role of preparatory processes when a reward is offered. Reward was also associated with reduced theta and beta power during task performance only. Razumnikova et al. (2009) found that alpha TRD versus baseline was of lesser magnitude under instructions to produce original solutions to the TTCT‐IF.

##### 3.3.2.3 Comparisons of high‐ and low‐creativity participants

In the alpha band, greater power (Jausovec, 2000; Sviderskaya, Taratynova, & Kozhedub, 2006) or reduced alpha TRD versus baseline (Petsche et al., 1997) was observed for artists compared to novices or for participants of high compared to low visual creativity, as measured by originality ratings of generated solutions. Volf et al. (2010a) reported opposite effects of creative ability in male and female participants. In males, high creativity (based on originality scores) was associated with greater upper alpha TRD compared to low‐creativity participants, while females of low creativity showed greater TRD compared to high‐creativity females. A tendency for greater TRD in posterior compared to anterior sites was also reported—in men, this was exhibited by high‐creativity individuals only in the lower and upper alpha bands. However, as Volf et al. (2010a) divided participants based on median splits of originality scores performed separately for males and females, it is unclear if originality scores were comparable between high‐ and low‐creativity men and women, and so differential effects of creativity in each group must be interpreted with caution.

In the theta and beta bands, Sviderskaya et al. (2006) observed greater power for art students compared to novices. These beta effects contrast with those of Molle et al. (1999), who reported greater beta power for individuals of low compared to high creativity.

##### 3.3.2.4 Trends across contrast types

After collapsing across the above contrast types, the percentage of studies reporting predominant power increases, decreases, or no clear effects differed across the delta, theta, alpha, and beta frequency bands (*p *=* *.016, Fisher's exact test). As only two studies reported gamma effects, these were excluded from this test, and the test collapsed across the lower and upper alpha bands, and separately, the lower and upper beta bands due to similar patterns in each. The observed effect reflected the observation that decreased power during visual creativity (vs. baseline, vs. control, original vs. standard solutions, high‐ vs. low‐creativity participants) was reported in the majority of studies examining effects in the delta (75%), theta (77.8%), and alpha (58.8%) bands, whereas in the beta band, most studies instead reported increased power (62.5%).

As specific predictions have been made regarding the role of the alpha band in creativity (see 1), we assessed whether the percentage of studies reporting predominant alpha increases, decreases, or neither differed according to the contrast type. For the lower and upper alpha bands, the distribution of outcomes differed across contrasts (*p*s < .001, Fisher's exact test)—100% of studies examining lower and upper alpha power during visual creativity versus baseline reported power reductions, while 75% of studies comparing high‐ and low‐creativity individuals instead reported greater power in the former group.

#### 3.3.3 Findings—Electroencephalography coherence

**Figure 4:**
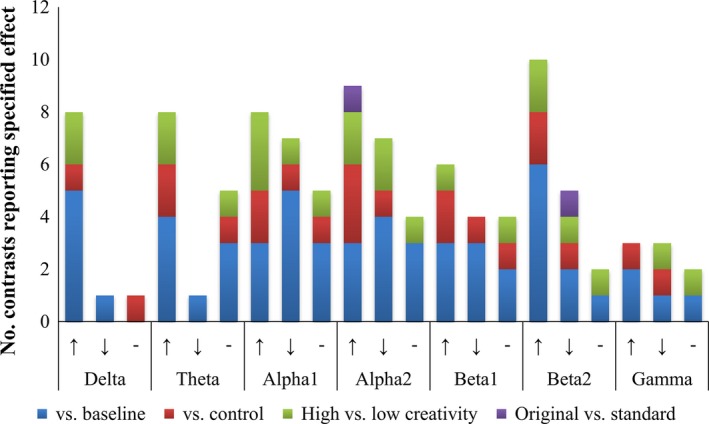
Summary of the frequency (number of contrasts showing relevant effect) with which studies reported predominant increases (↑), predominant decreases (↓), and no clear pattern of increases or decreases (−) in coherence in each frequency band. Findings of coherence changes during visual creativity versus baseline are displayed in blue; coherence changes versus control tasks in red; differences between high‐ and low‐creativity participants in green; and differences between production of original versus standard images in purple

The numbers of studies showing predominant coherence increases, decreases, or no clear pattern for the main contrast types are displayed in Fig. 4.

##### 3.3.3.1 Visual creativity versus baseline

For the low‐frequency delta and theta bands, a tendency toward widespread inter‐ and intrahemispheric coherence increases during visual creativity compared to baseline emerged (Bechtereva & Nagornova, 2007; Bhattacharya & Petsche, 2005; Kozhedub et al., 2007; Petsche, 1996; Razumnikova et al., 2009; Sviderskaya, 2011b). Petsche (1996) also noted some decreases in delta and theta frontal interhemispheric coherence. Volf, Tarasova, and Razumnikova (2010b) noted predominant increases in theta coherence in participants of high visual creativity, while low‐creativity participants showed predominant theta coherence decreases.

Findings were less consistent in the alpha range. For lower and upper alpha, roughly equal numbers of studies reported predominant coherence increases (Petsche, 1996; Sviderskaya, 2011a), decreases (Bechtereva & Nagornova, 2007; Kozhedub et al., 2007), and no clear pattern of coherence changes (Bhattacharya & Petsche, 2005; Razumnikova et al., 2009; see Fig. 4). Despite these inconsistencies, the foci of both inter‐ and intrahemispheric coherence increases and decreases were often at frontal sites, including long‐range intra‐ and interhemispheric coherence increases with frontal foci (Petsche et al., 1997; Sviderskaya, 2011a), and frontal interhemispheric decreases and increases (Petsche et al., 1997). Kozhedub et al. (2007) found that task‐related changes in coherence versus baseline were correlated between right frontal and right parietal sites. Volf et al. (2010b) reported predominant task‐related lower alpha coherence increases for high‐creativity participants and decreases for low‐creativity participants, indicating that individual differences in creativity or strategy use, in addition to task differences contribute to discrepancies between studies. Across both high‐ and low‐creativity groups, however, task‐related upper alpha coherence decreases were observed (Volf et al., 2010b).

Findings were again mixed for the lower beta range, with both predominant coherence increases (Bhattacharya & Petsche, 2005; Petsche, 1996; Sviderskaya, 2011a) and decreases (Bechtereva & Nagornova, 2007; Kozhedub et al., 2007), as well as findings of no clear pattern (Razumnikova et al., 2009; Volf et al., 2010b). The picture for upper beta was slightly clearer, with predominant coherence increases reported by six of nine studies (Bhattacharya & Petsche, 2005; Kozhedub et al., 2007; Petsche, 1996; Razumnikova et al., 2009, 2010; Sviderskaya, 2011a). Although Razumnikova et al. (2010) observed predominant coherence increases, some interhemispheric frontal coherence decreases were also reported. Two of the three studies examining gamma coherence versus baseline reported predominant increases (Bhattacharya & Petsche, 2005; Petsche, 1996; cf. Bechtereva & Nagornova, 2007).

##### 3.3.3.2 Visual creativity versus control

Sviderskaya (2011b) reported overall increases in delta and alpha coherence during visual creativity compared to a visual convergent thinking task. Bechtereva and Nagornova (2007) reported greater theta and alpha coherence during generation of original images from simple elements compared to generating images from memory. These effects differences consisted of widespread inter‐ and intrahemispheric increases, with foci at right frontal and, for alpha coherence, left parietal sites. Jausovec and Jausovec (2000) in contrast reported decreased alpha coherence during the TTCT‐IF compared to verbal creativity tasks. In the upper alpha band, these decreases were prominent between right frontal and bilateral parietal sites, although interhemispheric coherence increases were also observed between frontal sites. For the beta and gamma band, Bechtereva and Nagornova (2007) observed primarily left intrahemispheric coherence increases, with left temporal foci, alongside marked short‐ and long‐range interhemispheric coherence reductions.

In comparisons of visual versus verbal creative tasks, Razumnikova et al. (2009) reported increased theta coherence during the TTCT‐IF compared to a sentence generation task, while Razumnikova et al. (2010) observed greater upper beta coherence over frontal sites during the verbal remote associates task compared to the TTCT‐IF. During the TTCT‐IF, Razumnikova et al. (2009) reported increased upper beta coherence when generating original compared to standard figures. This was largely driven by right hemisphere intrahemispheric increases in female participants.

##### 3.3.3.3 Comparisons of high‐ and low‐creativity participants

Both studies examining effects of creative ability on delta coherence during visual creativity versus baseline reported greater coherence in high‐ compared to low‐creativity individuals (Bhattacharya & Petsche, 2005; Sviderskaya et al., 2006). Bhattacharya and Petsche (2005) found that these coherence differences were focused on posterior occipitotemporal sites, and took the form of inter‐ and intrahemispheric connections. A similar pattern of greater coherence in high‐ versus low‐creativity participants emerged in the theta band (Sviderskaya et al., 2006; Volf et al., 2010b), although Bhattacharya and Petsche (2005) reported no clear difference between groups in theta coherence.

For the lower alpha band, three of the four studies reported greater coherence in creative individuals (Jausovec, 2000; Sviderskaya et al., 2006; Volf et al., 2010b; cf. Bhattacharya & Petsche, 2005). Jausovec (2000) observed greater interhemispheric frontal coherence in high‐creativity participants, who also showed coherence increases versus baseline between bilateral frontal and midline parietal sites. Volf et al. (2010b) observed greater intrahemispheric coherence in high‐ versus low‐creativity participants due to the tendency of the former group to show increased intrahemispheric coherence versus baseline, while the latter showed reduced task‐related coherence versus baseline.

The picture was less consistent for the higher frequency ranges. For upper alpha, there were two reports of greater coherence in high‐ versus low‐creativity participants (Sviderskaya et al., 2006; Volf et al., 2010b), one of reduced coherence (Jausovec, 2000), and Petsche et al. (1997) reported no differences between groups. Bhattacharya and Petsche (2005) reported overall coherence reductions at frontal sites in artists versus novices across the alpha band.

Sviderskaya et al. (2006) and Bhattacharya and Petsche (2005) reported increased coherence in high‐ compared to low‐creativity participants across the beta range, although in the latter study this effect was restricted to right temporal sites, and reduced beta coherence was observed in artists versus novices over frontal sites. This study also provided the only examination of creative ability effects on gamma coherence, reporting reduced coherence over frontal sites in artists versus novices.

Sviderskaya (2011a) and Sviderskaya et al. (2006) examined effects of creative ability on *spatial synchronization* (SS) and/or *spatial disordering* (SD), measures of coherence based on linear and nonlinear relationships, respectively. Both found that artists compared to novices showed greater task‐related increases in coherence versus baseline, particularly over right frontal and occipital sites.

##### 3.3.3.4 Trends across contrasts

Collapsing across the four main contrast types, the percentages of studies reporting mainly coherence increases, decreases, or neither did not differ according to frequency band (*p *=* *.35, Fisher's exact test). There was no consistent trend toward coherence increases or decreases in any frequency band, aside from the delta band where 80% of studies reported increased coherence during visual creativity.

Visual creativity‐related effects on alpha coherence did not differ according to the contrast employed, for the lower or upper alpha band (*p *=* *.75; *p *=* *.86, Fisher's exact test).

#### 3.3.4 Hemispheric lateralization

No clear pattern of laterality of power or coherence effects emerged. Most studies examining EEG power effects reported bilateral effects, but Volf et al. (2010a) found that upper alpha TRD was greater at right temporal compared to left temporal sites. In contrast, Volf and Tarasova (2010) reported greater theta TRD in the left compared to the right hemisphere.

Bhattacharya and Petsche (2005) reported greater task‐related coherence increases in the right compared to the left hemisphere for the theta, alpha, beta, and gamma bands. For the measure of phase synchrony, this asymmetry was significant in artists but not novices, although an interaction of group with asymmetry was not directly assessed. Similarly, Razumnikova et al. (2009) reported greater intrahemispheric coherence in the right hemisphere for the lower theta, lower alpha, and upper beta bands. Contrasting with these findings, however, Kozhedub et al. (2007) reported a greater number of alpha coherence decreases versus baseline in the left compared to the right hemisphere.

## 4 Discussion

### 4.1 Overview of functional magnetic resonance imaging findings

Significant clusters revealed in the fMRI ALE meta‐analysis were in thalamus, right middle frontal, precentral, and inferior frontal gyri, left fusiform gyrus, left angular gyrus, and left cingulate gyrus. The ALE cluster in left fusiform gyrus, along with reported involvement in several studies of further bilateral occipitotemporal regions (e.g., inferior temporal gyrus, lateral occipital cortex), is consistent with predictions that visual creativity compared to control tasks is associated with greater visual processing, including visual imagery (e.g., Kosslyn & Thompson, 2003).

A recent meta‐analysis (Boccia et al., 2015) examined fMRI activity across studies of visuospatial creativity. This analysis, however, included only three of the six fMRI studies included here (Aziz‐Zadeh et al., 2013; Ellamil et al., 2012; Huang et al., 2013), and included foci for Kowatari et al.'s (2009) pen design versus fixation contrast despite the lack of significant visual design versus control task differences (see Section 3.2.2). Boccia et al. (2015) also included a study reporting coordinates during a task involving visual perception rather than active generation of visually creative solutions (Asari et al., 2008). Despite inclusion of additional studies (Gilbert et al., 2010; Park et al., 2015; Saggar et al., 2015), and stricter inclusion criteria, both meta‐analyses reported similar findings. Boccia et al. (2015) reported ALE clusters in bilateral thalamus, right middle (BA 6), and inferior (BA 9) frontal gyri that were in close proximity to regions reported in the current ALE meta‐analysis. The convergence between both meta‐analyses on similar regions of thalamus and right PFC provides additional support for the involvement of these or similar regions in visual creativity. Further supporting this, Gonen‐Yaacovi et al.'s (2013) meta‐analysis of fMRI studies of nonverbal creativity (including both musical and visual creativity) revealed significant ALE clusters in similar, but nonoverlapping, regions of right middle and inferior frontal gyri, left cingulate, and thalamus.

### 4.2 Overview of electroencephalography findings

Where visual creative tasks were compared against baseline fixation, the theta and lower and upper alpha frequency bands consistently showed reduced power, while higher frequency beta and gamma bands typically showed increased power relative to baseline. The theta band findings are at odds with reports that this frequency band typically shows increased power with increasing task demands (Klimesch, 1996; Klimesch, 1999). The studies reporting theta TRD (Razumnikova et al., 2009; Sviderskaya, 2011a; Volf & Tarasova, 2010) did not fully explain procedures for collecting baseline data (see Table S1; Section 4.5), and so it is possible that this result is due to lengthy baseline rest periods resulting in high levels of cognitive activity and thus theta power at baseline (Fink & Benedek, 2014). The findings of alpha TRD are consistent with a role of semantic and attentional processes during visual creativity (Klimesch, 2012). Increased beta power is indicative of increased alertness and active concentration during visual creativity (e.g., Gola, Kamiński, Brzezicka, & Wróbel, 2012; Klimesch, 1999). Such findings relative to fixation are, however, uninformative as to the neural mechanisms specific to visual creativity, as such changes may be observed in any task requiring greater cognitive resources than fixation.

Despite this, relatively few EEG studies directly compared activity during visual creativity and during appropriate control tasks, and those that did revealed largely inconsistent and contradictory findings in the alpha and beta bands. A contributing factor in these inconsistencies is likely the wide variety of control tasks employed, including reading, picture viewing, and generation of essays. The small number of studies that examined such effects in the delta and theta bands showed decreased power during visual creativity compared to control tasks, and equivalent comparisons revealed increased gamma power—the direction of these effects are consistent with the task versus baseline findings. Comparisons of participants of high‐ and low‐creative abilities revealed a consistent pattern of increased alpha power in the former group, although no clear pattern emerged for the remaining frequency bands. There was a tendency across studies for association of visual creativity with greater coherence in the theta, alpha, beta, and gamma bands compared to visual or verbal control tasks; and in high‐ compared to low‐creativity participants. This is indicative of increased functional connectivity during visual creativity, and in individuals of higher visual creativity ability (Fingelkurts, Fingelkurts, & Kähkönen, 2005; Thatcher, Krause, & Hrybyk, 1986).

### 4.3 Interpretation of findings in relation to accounts of the neural basis of creativity

#### 4.3.1 Prefrontal cortex functions

The proposed contribution of PFC functions to creativity has received consistent support from neuroimaging studies to date (e.g., Dietrich, 2004; Dietrich & Kanso, 2010; Gonen‐Yaacovi et al., 2013). Accounts of creative cognition have proposed a role of PFC‐mediated executive functions in creative idea generation, including updating of working memory, inhibition of irrelevant ideas, monitoring, and selection of generated solutions (Dietrich, 2004; Mumford et al., 2012). Previous reviews have reported involvement of bilateral inferior PFC, DLPFC, and MFG during a variety of visual and verbal creative tasks, although the precise PFC regions engaged differ according to task‐specific factors (Arden, Chavez, Grazioplene, & Jung, 2010; Dietrich & Kanso, 2010; Gonen‐Yaacovi et al., 2013). Effects relating to EEG coherence and power differences between verbal, visual, and musical creative and control tasks have often been observed to be focused on frontal sites (see Dietrich & Kanso, 2010; Fink & Neubauer, 2006; Jausovec & Jausovec, 2000; Petsche et al., 1997).

A contribution of PFC functions to visual creativity was supported in the current review. The ALE meta‐analysis revealed clusters in right MFG (BA 6/24) and IFG (BA 13/45), right precentral gyrus, and a left cingulate region extending into left medial PFC. The right IFG and MFG regions are consistent with recent meta‐analyses (Boccia et al., 2015; Gonen‐Yaacovi et al., 2013) supporting a role of these regions in nonverbal/visuospatial creativity (Section 4.1)—overlap was in fact observed between the right precentral cluster (BA 6), which extended into IFG, and Boccia et al.'s (2015) IFG (BA 9) cluster. The peak coordinates of the right middle frontal gyrus region (BA 6) revealed in the current meta‐analysis are identical to peak coordinates from Owen, McMillan, Laird, and Bullmore's (2005) meta‐analysis of n‐back working memory tasks, consistent with the proposed role of working memory, including the maintenance and manipulation of relevant information, in visual creativity (Oberauer et al., 2008). Similar right frontal regions to those revealed here have also been implicated in the suppression of unwanted or task‐irrelevant memories (Anderson et al., 2004; BA 6/13), which may engage similar mechanisms to the inhibition of irrelevant ideas in visual creativity.

Qualitative synthesis of all seven fMRI studies revealed PFC activity in left IFG (BA 44/45/47/11), left MFG (BA 6/9), including DLPFC (BA 46), and left SFG (BA 6/8), regions which were in close proximity to those reported in Gonen‐Yaacovi et al.'s (2013) meta‐analysis of 34 fMRI studies showing greater activity during nonverbal versus verbal creativity (e.g., left SFG: BA 6; left DLPFC: BA 46). Left DLPFC has been repeatedly associated with monitoring, inhibition, and selection and evaluation of solutions (e.g., Herd, Banich, & O'Reilly, 2006; Wagner, Maril, Bjork, & Schacter, 2001), and engagement of this region in the current meta‐analysis is consistent with the involvement of similar processes in the generation of visual creative solutions (Basadur, Graen, & Green, 1982; Mumford et al., 2012). An evaluative role of left DLPFC is supported by Ellamil et al.'s (2012) findings of greater activity in this region during the evaluation compared to generation phase of their book cover design task.

The reviewed EEG studies did not employ source localization, but the observation that visual creativity‐related coherence changes were often focused on frontal sites is again consistent with a contribution of frontal lobe functions (Dietrich, 2004). Foci of alpha and beta coherence increases included frontal sites in most studies examining this outcome (Bechtereva & Nagornova, 2007; Bhattacharya & Petsche, 2005; Jausovec & Jausovec, 2000; Sviderskaya, 2011b), indicative of increased functional connectivity between frontal regions and further cortical sites (Thatcher et al., 1986). This may involve top‐down modulation of downstream processes including perceptual, mnemonic, or attentive processes (e.g., Gazzaley et al., 2007). Petsche (1996) and Petsche et al. (1997) reported reduced interhemispheric frontal alpha coherence during visual creativity, interpreting this as increased independence of left and right PFC functions. Some studies also reported that task‐related power increases or decreases were particularly evident at frontal sites (Jaarsveld et al., 2015; Jausovec & Jausovec, 2000). Further behavioral and neuroimaging studies including EEG studies employing source localization in addition to appropriate control tasks are necessary to establish the subregions of PFC and associated cognitive processes contributing to visual creativity.

#### 4.3.2 Hemispheric lateralization

Mihov et al.'s (2010) meta‐analysis of EEG and fMRI studies of creativity reported right hemispheric dominance in visual and verbal creativity This, however, contrasts with other reviews and meta‐analyses (Dietrich & Kanso, 2010; Gonen‐Yaacovi et al., 2013; Wu et al., 2015) which reported no evidence of lateralization of creativity. Here, the ALE meta‐analysis of fMRI studies revealed activity in a number of bilateral PFC, inferior temporal, parietal, and subcortical regions. The PFC regions were primarily in the right hemisphere, with only the left cingulate cluster encompassing parts of left medial PFC. This contrasts with the qualitative findings, where more studies reported left (6/7) compared to right PFC (3/7) engagement. Together, these findings are indicative of greater consistency across studies and across tasks in the right PFC regions contributing to visual creativity, whereas left PFC regions, while commonly engaged, appear to vary according to task‐specific factors.

The apparent contribution of right PFC is consistent with Goel's (2014) Frontal Lobe Lateralization Hypothesis, which proposes that the right PFC, particularly DLPFC, supports ill‐structured representations that facilitate the open‐ended problem solving which is involved in visual design. Further studies directly assessing effects of hemisphere are, however, necessary to test the notion that right PFC regions (MFG, IFG) contribute to a greater extent to visual creativity than the corresponding left hemisphere regions. Two reviewed fMRI studies attempted comparison of effects in corresponding left and right PFC regions (Huang et al., 2013; Kowatari et al., 2009), but as they did not examine Hemisphere × Task/Group interactions, they fall short of providing direct evidence of lateralization (Section 3.2.3).

Of the 20 reviewed EEG experiments, only a minority reported effects of hemisphere (Section 3.3.4). Bhattacharya and Petsche (2005) observed greater task‐related coherence increases in the theta, alpha, and beta bands in the right compared to the left hemisphere, but Kozhedub et al. (2007) in contrast reported greater probability of task‐related changes in alpha coherence in the left compared to right hemisphere. In the majority of EEG studies, visual creativity‐related effects on power and coherence were largely bilateral, with no evidence of hemispheric dominance. No evidence was revealed of the alpha power asymmetry effects that have been associated with positive versus negative affect (Davidson, 1992) or response inhibition (Wacker, Chavanon, Leue, & Stemmler, 2010). In addition, the above studies reporting hemispheric effects did not assess whether these effects remained when comparing visual creativity to matched control tasks, and so it is unclear whether such effects are specific to visual creativity.

Taken together, the findings of the current review do not provide support for theories of hemispheric lateralization of visual creativity.

#### 4.3.3 Role of semantic and episodic memory processes

A number of the left‐lateralized regions identified in visual creativity contrasts in the reviewed fMRI studies have been implicated in semantic retrieval. A meta‐analysis of 120 functional neuroimaging studies (Binder, Desai, Graves, & Conant, 2009) found left MFG, SFG, and IFG and left inferior parietal lobe to be involved in semantic processing, regions which showed greater activity during visual creativity compared to control tasks in several of the reviewed fMRI studies (Aziz‐Zadeh et al., 2013; Ellamil et al., 2012; Huang et al., 2013; Park et al., 2015; Saggar et al., 2015). Left IFG, particularly pars orbitalis, has been consistently associated with semantic processing and retrieval (Binder et al., 2009; Liakakis, Nickel, & Seitz, 2011), and supports controlled access to conceptual representations (Badre, Poldrack, Paré‐Blagoev, Insler, & Wagner, 2005). This region showed greater activity during visual creativity compared to control tasks in several studies (Aziz‐Zadeh et al., 2013; Ellamil et al., 2012; Huang et al., 2013; Saggar et al., 2015), but was not identified in the ALE meta‐analysis. Activity during visual creativity in regions which have been linked to semantic processing does not directly support a role of the latter in visual creativity (Poldrack, 2006), but such a role is consistent with proposals that semantic retrieval and association are core components of creative ideation (Abraham & Bubic, 2015; Beaty et al., 2014; Mednick, 1962; Mumford et al., 2012).

Decreased EEG alpha power over frontal sites, particularly in the upper alpha band (~10–14 Hz) has been linked to semantic processing (Doppelmayr, Klimesch, Stadler, Pöllhuber, & Heine, 2002; Klimesch, 1999; Klimesch, Sauseng, & Hanslmayr, 2007; Klimesch, Schimke, & Pfurtscheller, 1993). Consistent with a role of semantic memory in visual creativity, widespread upper alpha power reductions which included frontal regions were observed in several studies compared to baseline (e.g., Molle et al., 1999; Nagornova, 2007; Petsche et al., 1997) and/or compared to control task performance (Jaarsveld et al., 2015; Jausovec & Jausovec, 2000). Upper alpha reductions were prominent over frontal sites in the latter two studies. For the critical contrast of visual creativity versus control tasks, however, this pattern was far from consistent across studies.

Episodic memory, memory for personally experienced events bound with context (Tulving, 1983), is thought to facilitate generation of creative ideas through a constructive process involving elements of previously experienced events (Benedek et al., 2014; Runco & Chand, 1995). Consistent with this, two of the reviewed fMRI studies of visual creative ideation (Ellamil et al., 2012; Park et al., 2015), in addition to studies of verbal creative ideation (e.g., Fink et al., 2009) reported greater activity during creative tasks in the hippocampus and parahippocampal cortex, regions strongly associated with mnemonic processing (e.g., Dickerson & Eichenbaum, 2010). The mediodorsal thalamic nucleus region revealed in the fMRI meta‐analysis has also been linked to recollection and familiarity in episodic memory (Zola‐Morgan & Squire, 1993; Zoppelt, Koch, Schwarz, & Daum, 2003), and is thought to relay inputs to and from hippocampal and prefrontal memory processing regions (Markowitsch, 1982; Xu & Sudhof, 2013).

#### 4.3.4 Visual imagery and visual processing

ALE meta‐analysis revealed activity for the contrast of visual creativity versus control tasks in the left fusiform gyrus. The majority (5/7) of the reviewed fMRI studies reported greater activity during visual creativity compared to control tasks in this and further occipitotemporal regions, including lateral and middle occipital cortex and middle and inferior temporal gyri (Aziz‐Zadeh et al., 2013; Ellamil et al., 2012; Huang et al., 2013; Park et al., 2015; Saggar et al., 2015). These findings are consistent with a greater role of processing of visual information during visual creativity. As the idea generation phase of each fMRI study involved visual input, whether verbal instructions (Gilbert et al., 2010; Saggar et al., 2015), images/image fragments (Aziz‐Zadeh et al., 2013; Huang et al., 2013; Kowatari et al., 2009), or sketches drawn by the participant (Ellamil et al., 2012), this activity may simply reflect perceptual and conceptual processing of visual input (Cowell, Bussey, & Saksida, 2010; Tyler et al., 2013). However, a further, not mutually exclusive possibility is that greater visual cortical activation is associated with greater engagement of visual imagery processes. Visual imagery engages many of the same or highly similar regions of occipitotemporal cortex as visual perception, including bilateral inferior and middle temporal gyri and middle occipital cortex (Ganis, Thompson, & Kosslyn, 2004; Ishai, Haxby, & Ungerleider, 2002), regions that were identified in several of the reviewed fMRI studies.

The left fusiform gyrus region revealed in the meta‐analysis has been repeatedly linked to visual imagery (Ganis et al., 2004; Kosslyn & Thompson, 2003). Consistent with suggestions that visual creativity, in particular visual design, engages manipulation of visual imagery, fMRI meta‐analyses have found overlapping left fusiform gyrus regions to be engaged in mental rotation (Tomasino & Gremese, 2015; Zacks, 2008). An overlapping region has also been implicated in retrieval of the semantic representations required to support visual imagery (Kan et al., 2003). The left lateralization of the observed fusiform activity is consistent with studies reporting that visual imagery predominantly engages the left hemisphere (D'Esposito et al., 1997; Sack, Camprodon, Pascual‐Leone, & Goebel, 2005), but as none of the reviewed studies formally compared effects in corresponding regions of left and right hemispheres, this account is not directly supported.

Several of the reviewed EEG studies reported that visual creativity‐related effects on power were larger or more significant over occipital compared to more anterior electrode sites, again consistent with a role of visual processing (e.g., Molle et al., 1999; Sviderskaya, 2011a; Sviderskaya et al., 2006; Volf et al., 2010a). Previous findings of reduced EEG power over occipitoparietal sites during visual imagery (Marks & Isaac, 1995; Salenius, Kajola, Thompson, Kosslyn, & Hari, 1995) were echoed by Jausovec and Jausovec (2000) who observed reduced lower and upper alpha power over occipital and left frontal sites only.

Task‐related coherence changes were often manifested by long‐range delta, alpha, and beta intrahemispheric coherence increases between frontal and posterior occipital sites, indicative of increased functional connectivity between these regions during visual creativity (Petsche, 1996; Sviderskaya, 2011b; Volf et al., 2010a). The apparent increases in frontal–posterior connectivity may reflect top‐down modulation of generation and manipulation of mental visual images (Mechelli, Price, Friston, & Ishai, 2004).

#### 4.3.5 Alpha frequency

Reduced task‐related alpha power (TRD) is thought to reflect increased cortical activation. Alpha suppression over frontal sites, particularly in the upper alpha band (~10–14 Hz) (Doppelmayr et al., 2002; Klimesch, 1999), has been associated with semantic processing (Klimesch et al., 1993, 2007), while lower alpha TRD has been associated with attentional processes (Klimesch et al., 2007; Section 3.3.1). Despite earlier conceptions of increased alpha power as “cortical idling” (Pfurtscheller, Stancak, & Neuper, 1996), it is now widely believed that alpha task‐related synchronization (TRS) reflects active processes including inhibition of task‐irrelevant processes, or internal processing demands (Fink & Benedek, 2014; Klimesch, 1999, 2012; Klimesch et al., 2007). This inhibitory control may contribute to creative task performance (e.g., Fink & Benedek, 2014; Grabner et al., 2007; Klimesch, Doppelmayr, & Hanslmayr, 2006; Sauseng et al., 2005). During both visual and verbal divergent thinking tasks, both increased (e.g., Fink, Grabner, Benedek, & Neubauer, 2006; Nagornova, 2007) and decreased (Jausovec & Jausovec, 2000; Razumnikova et al., 2009) alpha power has been reported. Dietrich and Kanso's (2010) systematic review of neuroimaging studies of creativity reported no clear pattern of alpha increases or decreases, either across verbal and visual divergent thinking studies, or across artistic and musical creativity studies. This echoes the current review, where in the few cases where EEG power during visual creativity tasks was directly compared with non‐rest control tasks, no clear pattern of increases or decreases in alpha power emerged. This suggests that depending on specific task demands or strategies, both semantic and attentional processing (TRD) and inhibitory processes (TRS) may be involved in visual creativity.

When visual creative tasks were compared to baseline fixation, a consistent pattern of lower and upper alpha power decreases was observed. This is consistent with greater cortical activation, and greater semantic and attentional processing during visual creativity versus fixation (e.g., Klimesch, 2012). Power changes versus baseline, however, provide a limited contribution to understanding of the neural basis of visual creativity, as they do not inform as to whether this response is specific to visual creativity (Arden et al., 2010)—similar patterns may emerge in response to any number of other tasks that are more cognitively demanding than fixation. Reduced alpha power compared to baseline has, for example, been elicited during working memory (Stipacek, Grabner, Neuper, Fink, & Neubauer, 2003), recognition (Dujardin et al., 1993), and visual classification (Pfurtscheller & Klimesch, 1990). Such findings along with early reports of reduced alpha power simply when eyes are open compared to closed (e.g., Klimesch, 1999) have consolidated the view that alpha suppression reflects cortical activation. Fink and colleagues have, however, consistently observed alpha power increases during verbal creative ideation (e.g., Fink & Neubauer, 2006; Fink et al., 2006), and a selective review by Fink and Benedek (2014) reported overall support for a role of alpha TRS in creative ideation. The majority of the evidence reported by Fink and Benedek (2014), however, also referred to studies of verbal ideation, and so these contradictory findings could be reconciled if inhibitory processes, manifested by alpha TRS, are more often engaged during verbal compared to visual creativity, the latter involving greater semantic and attentional processing.

Three of the four studies comparing alpha power in participants of high and low creativity reported increased lower and upper power in the former group. However, due to the small number of studies and as these increases reflected both reduced TRD (Petsche et al., 1997) and increased absolute power (without reference to baseline; Jausovec, 2000; Sviderskaya et al., 2006), it is difficult to arrive at a clear interpretation of this finding in relation to accounts of the role of alpha TRS/TRD. Furthermore, as some studies divided participants into high‐ and low‐performance groups via a median split based on originality of generated solutions, high‐ and low‐creativity groups may not have demonstrated comparable creative ability across studies.

A further caveat is that few studies directly compared visual creative tasks with non‐rest control tasks, and of those that did, findings were inconsistent for alpha power and coherence. To form clearer conclusions on the contributions of alpha oscillations to visual creativity, a greater number of quality studies (see Sections 4.4 and 4.5) employing comparable contrasts, tasks and measures are necessary.

### 4.4 Methodological issues in reviewed studies

The qualitative synthesis of EEG studies revealed relatively few consistent findings, and despite several significant clusters emerging in the ALE meta‐analysis, findings of the fMRI studies also differed substantially. This lack of consistency may stem from substantial heterogeneity in the visual creative and control tasks, contrasts conducted, and outcome measures recorded (see Amabile, 1983). Even where the same creative task was employed, for example, TTCT‐IF, it was compared against a variety of control tasks, ranging from simple line tracing to more cognitively demanding visual and verbal problem‐solving and memory tasks. Evidence of a common neural or electrophysiological basis of visual creativity may be obscured by comparisons against tasks eliciting widely differing cognitive processes (Arden et al., 2010).

Tasks also differed in their focus, with visual design tasks highlighting the functionality of generated solutions (e.g., Ellamil et al., 2012; Gilbert et al., 2010; Kowatari et al., 2009); artistic tasks emphasizing esthetics (e.g., Bhattacharya & Petsche, 2005; Petsche, 1996); and others emphasizing the originality or fluency of solutions (e.g., Kozhedub et al., 2007; Volf et al., 2010a). Greater consistencies in the neural or electrophysiological correlates of visual creativity may be detectable by subdividing studies according to these goal‐related factors, that is, tasks requiring generation of solutions that are (1) functional, (2) esthetically pleasing, or (3) original. However, heterogeneity in procedures, populations studied, contrasts conducted, and outcome measures recorded meant that such subdivisions were unfeasible here due to low numbers of comparable studies within each category.

A further key issue is that of the timing and duration of sampling of neural activity associated with visual creativity. Most reviewed studies recorded and averaged neural activity across the duration of the visual creativity task, but in a subset of studies (e.g., Aziz‐Zadeh et al., 2013; Gilbert et al., 2010; Jaarsveld et al., 2015) participants were asked to signal when the task was complete, and activity was averaged from the start of the task until the response. Both methods are likely to capture the cognitive and neural processes involved in visual creative ideation, and likely also idea evaluation, but due to the long sampling periods (typically ~30 s) are likely also to include further cognitive processes both related and unrelated to visual creativity, for example, comprehension of task instructions, maintenance of visual representations, default mode activity (Fink, Benedek, Grabner, Staudt, & Neubauer, 2007; Fox & Raichle, 2007), potentially reducing the signal to noise ratio, and ability to detect processes specific to visual creativity (Abraham, 2013).

### 4.5 Quality assessment

Quality assessment of the reviewed studies (Section 2.2, Table S1) revealed that most did not meet all quality criteria. Many did not provide complete descriptions of participant selection and demographic information (41%), task procedure (33%), neuroimaging procedure and outcome measures (7%), and analyses and results (15%). This not only precludes replication, but also leads to difficulties in directly comparing findings across studies (Whiting et al., 2003). A further critical issue is that 37% of the 27 experiments did not conduct appropriate multiple comparisons corrections, or in the case of EEG studies, correction for violation of sphericity, limiting the reliability of reported findings.

Lack of controls in 48% of experiments of factors such as task difficulty or duration between experimental and control tasks (e.g., Jausovec, 2000; Nagornova, 2007) introduced further potential confounds. In 60% of EEG studies, visual creativity was simply compared against baseline fixation/rest and not a matched non‐rest control task, leading to the inability to infer whether effects are specific to visual creativity or are observed during multiple cognitive processes. Another difficulty in synthesizing results across EEG studies stemmed from differences in outcome measures—several reported differences in raw measures of power, while others reported task‐related power corrected for baseline power.

### 4.6 Future directions

To more clearly establish the neural basis of visual creativity, it is necessary to address the above methodological issues and ensure the quality criteria outlined in Section 2.2 are met. It is important to introduce measures to ensure control of confounds between visual creative and control tasks (Abraham, 2013). Greater standardization of the control tasks employed or use of several control tasks within the same sample will better enable identification of commonalities in neural activity between studies and between visual creative tasks. In fMRI studies, examination of functional overlap between regions identified in contrasts of visual creativity against multiple appropriate control tasks would enable identification of regions that are reliably engaged in, and are specific to, visual creativity.

It is also important to acknowledge that visual creativity is a composite, nonunitary construct and likely consists of multiple distinct cognitive and neural processes (Dietrich & Kanso, 2010)—a common neural basis may not be readily detectable. The mechanisms underlying visual creativity may differ according to task‐specific features such as focus on (1) functionality, (2) originality and/or fluency, and (3) esthetics of produced visual solutions. Most studies, particularly EEG studies, have thus far employed tasks emphasizing originality or fluency, and there remain insufficient comparable studies (in terms of procedures and outcome measures) within each proposed type of visual creative task for reliable comparisons across studies. As a result, quantitative and qualitative syntheses collapsed across these task divisions in the current review. The meta‐analysis findings, which incorporated studies employing tasks focusing on the functionality and originality/fluency of solutions, in addition to one study with no clear task focus, however, offer promising evidence that certain regions, including fusiform gyrus, thalamus, and right PFC, contribute to visual creativity across multiple task foci.

An aim of this review was to assess evidence for a consistent neural/electrophysiological basis of creativity when focusing on the visual domain only, and only on active generation of visual creative forms. It was hoped that this would lead to greater clarity of interpretation and consistency of findings compared to previous reviews which have sought a common neural basis across multiple domains of creativity (visual, musical, verbal) and across insight problem solving, perception/memory of existing creative forms in addition to their active generation (e.g., Dietrich & Kanso, 2010; Gonen‐Yaacovi et al., 2013). However, it is important to consider how findings in the visual domain relate to those from and across other domains of creativity by assessing the extent to which creative tasks exhibit shared variance in terms of cognitive and neural contributions.

The current meta‐analysis revealed little evidence of overlap in the cortical regions engaged compared to Boccia et al.'s (2015) meta‐analyses of musical and verbal creativity, aside from an overlapping region of left medial frontal gyrus (BA32) here and in the musical creativity meta‐analysis. This may be due to lack of power in the current meta‐analysis due to small numbers of studies, but also reinforces Boccia et al.'s (2015) findings of domain‐specific as well as domain‐general cortical contributions to creativity. However, to directly contrast visual creativity with other forms of creative ideation it will be necessary for future studies to directly compare visual and nonvisual creativity within the same participants. A small number of the reviewed EEG studies reported power and/or coherence effects versus baseline of similar magnitude and in the same direction for both visual and verbal divergent thinking (Jausovec & Jausovec, 2000; Molle et al., 1999; Razumnikova et al., 2009, 2010). However, as these findings refer to baseline contrasts, comparable effects may be observed with any number of tasks requiring cognitive effort (Section 4.3.5).

Consistent with Arden et al.'s (2010) suggested psychometric approach to creativity, given suggestions that visual creativity relies on semantic, executive, and visual imagery processes, these claims could be evaluated by assessing whether ability in these cognitive domains predicts visual creative ability, or neural activity elicited during visual creativity. Such associations could be compared across multiple domains of creativity and across task foci.

Machine learning algorithms (see Brouwer, Zander, van Erp, Korteling, & Bronkhorsst, 2015; Mwangi, Tian, & Soares, 2014, for reviews) offer promising avenues in identification and classification of EEG and fMRI features associated with visual creativity compared to control tasks, or in classification of features associated with visual creativity emphasizing functionality, esthetics, and originality. In fMRI, multivariate pattern recognition algorithms may aid in identifying not only which cortical regions show involvement in visual creativity, but also which regions show evidence of representing visually generated creative ideas, and which regions differentiate between the generation of functional, esthetic, and original visual solutions (Mur, Bandettini, & Kriegeskorte, 2009).

The inherent difficulty in temporal isolation of the processes directly relevant to creativity has been noted (Abraham, 2013), leading Fink et al. (2007) to suggest a method via which participants indicate the moment of idea generation, and the activity immediately preceding the button press is examined. The issue of selection of an arbitrary sampling duration is not fully avoided using this method, but in future studies, adoption of a common method of isolating activity associated with creative ideation will aid comparability of findings across studies.

These suggestions for future research are summarized below:

1. Ensure greater between‐study consistency in the nature of creative and control tasks employed, and adequate control of confounds between creative and control tasks.
2. Directly examine effects of task focus (e.g., function, esthetics, originality) on the neural basis of visual creativity.
3. Directly contrast and compare the neural and cognitive basis of visual compared to verbal and musical creativity (Arden et al., 2010).
4. Capitalize on advancements in machine learning and multivariate pattern analysis techniques to identify features associated with representation of visual creative ideas.
5. Employ standard methods across studies of isolating the time period to be examined, for example, following Fink et al.'s (2007) approach of examining neural activity directly preceding pressing of an “idea button.”

## 5 Conclusions

Meta‐analysis of six fMRI studies revealed, across studies, greater activity in regions of right middle and inferior frontal gyri during visual creativity compared to non‐rest control tasks, and EEG power and coherence effects during visual creativity were often focused on frontal sites. These findings are consistent with theories of creative cognition that propose an integral role of PFC functions including working memory, inhibition of task‐irrelevant information, selection among competing representations, and monitoring and evaluation of solutions. Meta‐analysis of fMRI studies and qualitative synthesis of fMRI and EEG studies also supported a role of occipitotemporal regions in visual creative task performance, consistent with a role of increased visual processing, including visual imagery and visual image manipulation, during visual creativity. Neither fMRI nor EEG studies provided clear support for the notion of right hemispheric dominance in visual creativity, although the meta‐analysis findings demonstrated greater cross‐study consistency in the right compared to left PFC regions engaged. Synthesis of the EEG studies did not provide consistent support for suggestions that either increases or decreases in alpha power contribute to visual creativity.

## Funding information

This study was supported by the Engineering and Physical Sciences Research Council (Grant/Award Number: EP/M012123/1 and EP/M01214X/1).
