# Supplementary material for: Functional neuroimaging of visual creativity: a systematic review and meta‐analysis
Source: Brain Behav. 2016 Aug 11;6(10):e00540. doi: 10.1002/brb3.540 (PMC5064346; doi:10.1002/brb3.540)
Supplement: Supplementary file 1 [file BRB3-6-e00540-s001.docx]

**Supplementary Table S1. Summary of quality assessment criteria met by each study.** Quality criteria were: A) Clear description of participant selection criteria and relevant demographic information; B) Visual creativity task compared against an appropriate control task; C) Sufficient detail on the task procedure to ensure reproducibility; D) Sufficient detail on the neuroimaging procedure and outcome measures to ensure reproducibility; E) Sufficient information on analyses and results for reproducibility; F) Conclusions are justified based on the analyses conducted e.g., appropriate multiple comparisons corrections; G) No substantial confounds between groups or conditions. PFC = prefrontal cortex; ROI = region of interest.

| Study | A | B | C | D | E | F | G | % | Notes |
| --- | --- | --- | --- | --- | --- | --- | --- | --- | --- |
| *fMRI studies* | | | | | | |  |  |  |
| Aziz-Zadeh et al., 2013 | 1 | 1 | 1 | 1 | 0 | 1 | 0 | 71 | Number of movements of shapes not controlled between creative and control tasks; longer completion time for creative vs. control task; test statistics and coordinates not reported for creative and control vs. rest so cannot determine if functional overlap |
| Huang et al., 2013 | 1 | 1 | 1 | 1 | 0 | 0 | 0 | 57 | Longer completion time for creative vs. uncreative task. Creative vs. uncreative only significant using Small Volume Correction. Results not reported for tasks vs. fixation. No information on multiple comparisons correction. No coordinates for PFC ROIs. Hemisphere* Task not tested for in ROIs can't reliably say different effect of task in each hemisphere |
| Park et al., 2015 | 0 | 1 | 1 | 1 | 1 | 1 | 1 | 86 | Unclear whether fMRI conducted for all participants or only high and low schizotypy scorers |
| Ellamil et al., 2012 | 1 | 1 | 1 | 1 | 1 | 1 | 0 | 86 | Results for Generate and Evaluate vs. Trace not fully reported. More writing in Evaluation than Generate (but regressing out number of words didn't alter findings) |
| Gilbert et al., 2010 | 1 | 1 | 1 | 1 | 1 | 1 | 0 | 86 | Functional connectivity findings did not survive multiple comparisons correction so are not reviewed. Creative ideation not separated from reading of instructions; difficulty of creative and control tasks not controlled. |
| Kowatari et al., 2009 | 0 | 1 | 1 | 1 | 1 | 0 | 0 | 57 | No differences between design and counting task or between experts and novices. Effects examined only in ROIs, precise location (coordinates) not stated. Cannot reliably infer different effects in experts and novices as group not included as a factor. |
| Saggar et al., 2015 | 1 | 1 | 1 | 1 | 1 | 1 | 0 | 86 | Number of moves during drawing not controlled for and creativity scores higher with greater numbers of elements within a picture - suggests confound of movement in analyses of relationship between activity and creativity. Linguistic processing required in creative but not control task. |
| *EEG studies* | | | | | | |  |  |  |
| Bechtereva & Nagornova, 2007 | 0 | 1 | 0 | 1 | 1 | 1 | 1 | 71 | Minimal information on task procedure, e.g., timings |
| Bhattacharya & Petsche, 2005 | 1 | 0 | 0 | 1 | 1 | 1 | 0 | 57 | Differences in EEG measures at rest not assessed. No control task, only fixation. Apparent between-group differences in age and EEG duration |
| Jaarsveld et al., 2015 | 1 | 1 | 0 | 1 | 1 | 1 | 1 | 86 | Full details of task procedure not provided |
| Jausovec, 2000 (Experiment 2) | 0 | 0 | 1 | 1 | 1 | 1 | 0 | 57 | Duration and difficulty of tasks not controlled; task order not counterbalanced |
| Jausovec & Jausovec, 2000 (Experiment 2) | 1 | 0 | 1 | 1 | 1 | 1 | 0 | 71 | No counterbalancing of task order |
| Kozhedub et al., 2007 | 0 | 0 | 0 | 1 | 1 | 1 | 0 | 43 | Most images produced were classed as standard (65%). 38% of participants didn't produce any original images. |
| Molle et al., 1999 | 1 | 1 | 1 | 1 | 1 | 0 | 1 | 86 | No between-groups multiple comparisons correction |
| Nagornova, 2007 | 0 | 0 | 0 | 1 | 1 | 1 | 0 | 43 | Creative and control tasks differed in difficulty and strength of emotions induced |
| Petsche, 1996 (Experiment 2) | 0 | 0 | 0 | 1 | 1 | 0 | 1 | 43 | Control and creative tasks not directly compared; no multiple comparisons correction; |
| Petsche et al., 1997 (Exp. 1) | 0 | 0 | 0 | 1 | 1 | 0 | 1 | 43 | No information on multiple comparisons correction, no direct comparisons between groups or between creative and control tasks |
| Petsche et al., 1997 (Exp. 3) | 0 | 0 | 0 | 0 | 0 | 0 | 1 | 14 | Insufficient information on procedure, results, analyses for reproducibility |
| Razumnikova et al., 2009 | 0 | 1 | 1 | 1 | 1 | 1 | 1 | 86 |  |
| Razumnikova et al., 2010 | 1 | 1 | 1 | 1 | 1 | 1 | 1 | 100 |  |
| Sviderskaya, 2011a | 0 | 1 | 0 | 0 | 0 | 0 | 1 | 29 | Insufficient information on multiple comparisons corrections, procedure and analyses for reproducibility |
| Sviderskaya, 2011b | 1 | 0 | 1 | 1 | 1 | 0 | 1 | 57 | No direct between group comparisons |
| Sviderskaya et al., 2006 | 1 | 0 | 1 | 1 | 1 | 0 | 1 | 71 | Interpretation refers to between group differences, but these only directly examined in limited contrasts |
| Volf et al., 2010a | 1 | 0 | 1 | 1 | 1 | 1 | 1 | 86 | Effects of instructions not directly compared |
| Volf & Tarasova, 2014 | 1 | 1 | 1 | 1 | 1 | 1 | 0 | 86 | Unequal trial numbers between participants because number of presentations depends on idea generation rate. Difference in trial numbers between conditions not tested. No counterbalancing of conditions. |
| Volf & Tarasova, 2010 | 1 | 1 | 1 | 1 | 1 | 0 | 1 | 86 | Sex*Originality*Electrode for theta2 sync treated as significant, but did not survive Greenhouse-Geisser correction |
| Volf et al., 2010b | 1 | 0 | 1 | 1 | 1 | 1 | 0 | 71 | Median split of originality scores performed separately for men and women - unclear if scores comparable between low and high originality men and women |
